# Hydrolysis-based degradation of organophosphorus nerve agents and simulants: water/base-dependent reactivity, catalytic materials and life-cycle considerations

**DOI:** 10.1039/d6ra04543j

**Published:** 2026-08-06

**Authors:** Ji-Won Kwak, Jong Beom Lee, Byeong-Chan Park, Jun-Ho Cha, Sang Goo Jeon, Geon-Hee Kim, Young Durk Park, Kyeongseok Oh, Joo-Il Park

**Affiliations:** a Department of Chemical & Biological Engineering, Hanbat National University Daejeon 34158 Republic of Korea jipark94@hanbat.ac.kr +82-42-821-1530; b Climate Change Research Division, Korea Institute of Energy Research Daejeon 34129 Republic of Korea; c Department of Mechanics-Materials Convergence System Engineering, Hanbat National University Daejeon 34158 Republic of Korea; d Department of Chemical & Biological Engineering, Inha Technical College Incheon 22212 Republic of Korea

## Abstract

This review presents field-relevant criteria for selecting hydrolysis-based decontaminants for organophosphorus nerve-agent decontamination according to water and base availability. We compare metal oxides, zeolites and porous silicas, carbon-based composites, metal–organic frameworks (MOFs), and polyoxometalates (POMs) by active-site structure, medium dependence, reaction selectivity, and product-induced deactivation. Because reported half-life and rate constants depend strongly on reaction conditions, we reclassify literature data using water availability (W-Class) and base availability (B-Class) and relate them to dry surfaces, humid interfaces, and bulk aqueous systems. This classification clarifies how apparent catalytic performance changes with water supply, base or buffer addition, interfacial mass transfer, and product accumulation. Here, life-cycle considerations are scoped as screening-level cradle-to-gate production burdens, complemented by W/B-Class-linked discussion of operational resource inputs, secondary-waste generation, spent-solid handling, and spent-liquid management. Therefore, this review proposes that hydrolysis-based decontaminant selection should move beyond laboratory rate comparisons and toward system-level assessment based on agent identity, water/base availability, reaction selectivity, application form, cradle-to-gate production burden, and operational waste-management requirements.

## Abstract 2

This review presents field-relevant criteria for selecting hydrolysis-based decontaminants for organophosphorus nerve-agent decontamination according to water and base availability.

## Graphical abstract

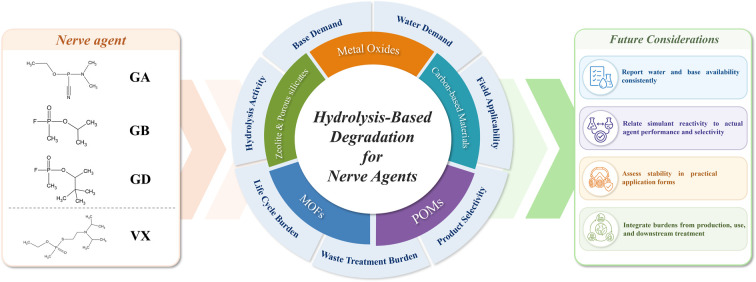

## 1. Introduction

Chemical warfare agents (CWAs) are highly hazardous toxic chemicals that disrupt physiological functions or cause fatal injury, posing serious threats in intentional-release and emergency-response scenarios.^1,2^ Although the international community adopted the Chemical Weapons Convention (CWC) in 1993, which entered into force in 1997 to prohibit such weapons of mass destruction,^1^ nerve agents (NAs) remain among the most concerning classes of CWAs because their high acute toxicity can cause mass casualties within a short time.^3^ After release, NAs can also contaminate exposed surfaces, soils, porous materials, and water streams, creating environmental decontamination and waste-management challenges.^4,5^

**Fig. 1:**
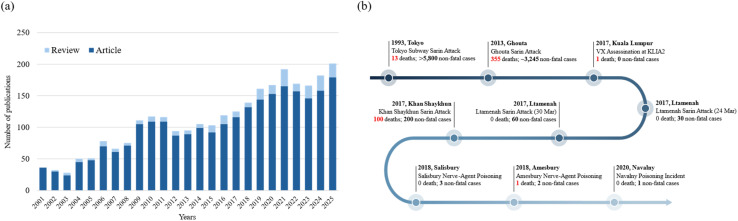
(a) Annual scopus publications retrieved from 2001 to 2025, categorized as articles and reviews (keywords: nerve agent, chemical warfare agent, organophosphorus nerve agent, nerve agent simulant and chemical warfare agent simulant.) (b) historical incidents related to nerve agents since 1995 (constructed from OPCW and UN reports and the cited literature; ref. 6–15.).

Fig. 1 demonstrates the need for continued research on NAs from two perspectives. Fig. 1(a) shows the annual number of publications retrieved from Scopus using the keywords “nerve agent,” “chemical warfare agent,” “organophosphorus nerve agent,” “nerve agent simulant,” and “chemical warfare agent simulant.” The figure indicates that a broad body of research on NAs, including studies on toxicity, protection, simulants, and decontamination, has steadily accumulated since the early 2000s, suggesting that NAs have long been recognized as a challenge requiring sustained scientific and technological responses. Meanwhile, Fig. 1(b) presents major NA-related incidents reported since 1995 in chronological order. It shows that such incidents were not fully eradicated even under the CWC regime, but have persisted in various forms, including urban mass exposure and civilian exposure events.^6–15^ This persistence is consistent with the steady growth of the related literature shown in Fig. 1(a), indicating that NAs continue to require sustained research on toxicity, protection, decontamination, and environmental management.

Accordingly, developing effective decontaminants for CWAs remains an important challenge. Various approaches have been proposed to address this issue, including hydrolysis, chlorine-based oxidation, advanced oxidation processes (AOPs), and catalytic or photocatalytic systems.^2,16–20^Table 1 summarizes recent review articles published between 2019 and 2024 on CWA decontamination, with particular emphasis on NAs.

**Table 1:** Recent review papers on CWA decontamination published from 2019 to 2024

Authors	Year	Decontaminants	Reviewed contents	CWAs	Ref.
Labaška *et al.*	2024	Hydrolysis; chlorine-based decontaminants; AOPs; MOFs; POMs; zeolites; reactive polymers	Decon strategies	Vesicants (HD; HN1–HN3; Lewisite); nerve agents (GA, GB, GD, VX)	16
Couzon *et al.*	2022	Porous textile composites: activated carbon/ACFs; functionalized ACFs; zeolites; MOFs; aerogels	Porous textile and porous composites	CWAs (vesicants, nerve, blood, choking agents) and simulants	17
Zammataro *et al.*	2020	Metal catalysts; polymeric catalysts; heterogeneous catalysts; enzymes; MOFs	Hydrolysis catalysis	Nerve agents (G-, V-, A-type), simulants	18
Kirlikovali *et al.*	2020	Zr-MOFs (solution & textile-coated implementations)	Zr-MOF hydrolysis	Organophosphorus substrates, nerve-agent simulants	19
Phadatare *et al.*	2019	Zr-based and Cu-based MOFs integrated into fibers/fabrics	MOF textiles	Nerve agents and other CWAs	20

Labaška *et al.* reviewed CWA decontamination strategies from a broad materials perspective, including metal–organic frameworks (MOFs), polyoxometalates (POMs), zeolites, and reactive polymers. They compared the strengths and limitations of each material class and highlighted the potential of complementary combinations for safer and more sustainable decontamination.^16^ Zammataro *et al.* provided an in-depth analysis of chemical hydrolysis mechanisms across diverse catalyst classes, including metals, polymers, and enzymes.^18^ Kirlikovali *et al.* systematically discussed the design principles and mechanisms of Zr-MOF-based catalysts for NA hydrolysis, with emphasis on their engineering implementation in protective equipment and coatings.^19^ Meanwhile, Couzon *et al.* reviewed decontamination-functional textiles incorporating porous materials such as MOFs and aerogels,^17^ whereas Phadatare *et al.* focused on the fabrication and applications of MOF-functionalized fabrics.^20^

Overall, these studies have provided an important scholarly basis for CWA decontamination by reviewing its mechanisms and material platforms. However, hydrolysis rates and product distributions are governed not only by the intrinsic activity of the decontaminant but also by operational conditions such as water availability, base availability, and contaminant phase behavior.^18–23^ Thus, even the same decontaminant may require different conditions to perform effectively in bulk aqueous solutions, wet surfaces, or moisture-limited solid interfaces. Literature-reported reaction data should therefore be reinterpreted in the context of actual decontamination environments, including contaminated surfaces, porous media, and aqueous waste streams. This approach can support the selection of decontaminants optimized for specific water/base regimes and help identify candidates with high field and environmental applicability.

In this context, this review focuses on organophosphorus nerve agents (OPNAs), a class of CWAs with shared phosphorus (P)-centered reactivity, and reexamines hydrolysis as a detoxification route based on bond cleavage at the P center. For VX, both degradation kinetics and suppression of the highly toxic byproduct EA-2192 must be considered.^22,24^ Accordingly, we evaluate decontaminant performance in relation to reaction-engineering variables, including water and base availability, reaction selectivity, and product-induced catalyst deactivation. We assess production-stage burdens within a screening-level cradle-to-gate LCA framework. Because a full cradle-to-grave LCA lies beyond the scope of this review, we discuss use-phase and post-treatment burdens qualitatively across W/B-Class regimes, focusing on water and base inputs, spent-solid handling, spent-liquid recovery, and waste-management requirements. Together, these analyses provide an integrated basis for comparing field applicability, operational resource requirements, product selectivity, and environmental burdens.

## 2. Classification of nerve agents

**Fig. 2:**
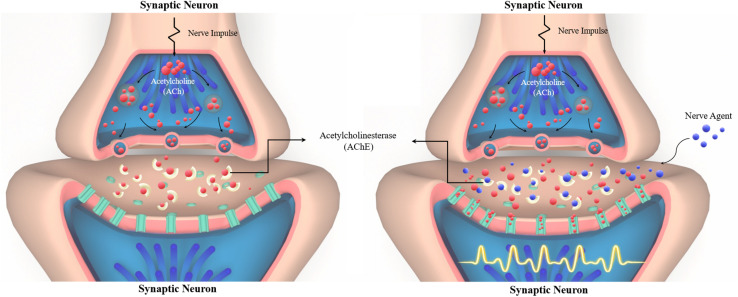
Schematic illustration of NA-induced AChE inhibition and ACh accumulation.

NAs are among the most acutely toxic classes of CWAs, causing severe physiological injury by disrupting signal transmission in the human nervous system.^25^ Most NAs are organophosphorus compounds (OPCs) and share a common toxic mechanism: irreversible inhibition of acetylcholinesterase (AChE), the enzyme that hydrolyzes the neurotransmitter acetylcholine (ACh). Upon exposure to NAs, the agent's P center reacts with the serine residue in the active site of AChE through phosphylation, forming a covalent adduct and inactivating the enzyme. This inhibition causes ACh to accumulate in the synaptic cleft, sustaining nerve stimulation and producing fatal symptoms such as convulsions, respiratory distress, and paralysis. Fig. 2 summarizes this toxicity pathway from AChE inhibition to ACh accumulation.

In particular, aging after formation of the NA-AChE adduct is a key factor that determines the urgency of initial decontamination. Aging involves dealkylation of the phosphylated enzyme adduct, which stabilizes the anionic charge at the active site and markedly limits subsequent enzyme reactivation by oxime-based therapeutics.^26,27^ The aging rate depends strongly on agent structure: soman (GD) ages rapidly within minutes, whereas sarin (GB) and VX age more slowly, typically over hours and tens of hours, respectively.^27^ Thus, agent-specific aging behavior provides an important basis for defining the effective response window after exposure and selecting initial decontamination strategies.

**Fig. 3:**
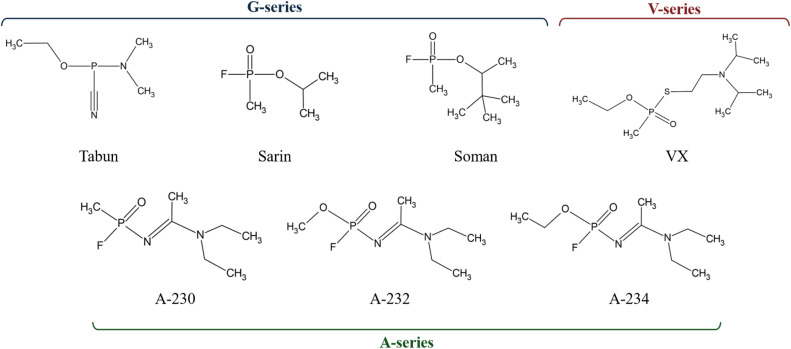
Chemical structures and classification of representative G-, V-, and A-series NAs.

The molecular structures and physicochemical properties of NAs govern their environmental behavior and primary exposure routes.^4,25,27^ Highly volatile agents with high vapor pressures tend to act mainly through respiratory inhalation, whereas agents with lower volatility and higher lipophilicity have greater potential for dermal absorption and secondary contamination through surface persistence.^4,5,26–30^ Water solubility and hydrolytic stability also differ among agents; therefore, degradation rates and residual toxicity can vary substantially even under the same decontamination conditions.^4,22^ On the basis of these structural features, NAs can be broadly classified into G-series, V-series, and A-series (Novichok) agents (Fig. 3).

### 2.1. G-series, V-series and A-series

NAs are broadly classified into the G-, V-, and A-series based on their historical origin and chemical structural features. Table 2 summarizes representative physicochemical properties of major NAs, including vapor pressure, aqueous solubility, and density.

**Table 2:** Physicochemical properties of representative NAs

Nerve agent	Vapor pressure (mmHg, 25 °C)	Water solubility (g L^−1^, 20–25 °C)	Density (g mL^−1^, 20–25 °C)	Ref.
Tabun (GA); ethyl *N*,*N*-dimethylphosphoramidocyanidate	0.057–0.070	98	1.077	31
Sarin (GB); isopropyl methylphosphonofluoridate	2.7–2.9	Miscible	1.09	28 and 32
Soman (GD); pinacolyl methylphosphonofluoridate	0.40	21	1.02	29
VX; ethyl (2-bis(propan-2-yl)aminoethyl)sulfanyl)(methyl)phosphinate	0.0007	30	1.01	5 and 33
A-230; *N*-(1-(diethylamino)ethylidene)-*P*-methylphosphonamidicfluoride	0.0160	4.823	1.612	34 and 35
A-232; methyl *N*-1-(diethylamino)ethylidenephosphoramidofluoridate	0.0111	1.775	1.515	34 and 35
A-234; ethyl *N*-1-(diethylamino)ethylidenephosphoramidofluoridate	0.0128	0.651	1.414	34 and 35

G-series NAs are first-generation agents synthesized in Germany during the 1930s and 1940s, with GA, GB, and GD as representative examples.^25,28–31^ They share a central phosphoryl (P=O) group and contain a fluoride or cyanide leaving group. Their high vapor pressures promote vapor-phase dispersion and rapid intoxication through respiratory exposure.^27–29^ In particular, GB combines high vapor pressure with high aqueous solubility. Thus, although GB can undergo hydrolysis under aqueous conditions, its rapid airborne dispersion increases the risk of mass exposure.^28^ GD is somewhat less volatile than GB but remains highly dispersible. The GD-AChE adduct undergoes rapid aging, which quickly converts the inhibited enzyme into an oxime-resistant form and sharply limits the time available for AChE reactivation.^26,29^

V-series agents are second-generation NAs developed in the United Kingdom in the 1950s during pesticide research, with VX as the representative compound.^5,25^ They generally share a methylphosphonothioate structure containing a P–S bond and a bulky aminoalkyl thio substituent, which gives them much lower vapor pressures and more limited volatility than G-series agents.^27^ VX is a colorless, odorless, viscous liquid with extremely low vapor pressure at room temperature. It therefore does not readily evaporate into air and can persist on surfaces or in soil, increasing the risk of dermal and contact exposure.^5^ Although VX has limited but measurable aqueous solubility, its hydrolysis in aqueous environments depends strongly on pH, temperature, and the water matrix, with reported half-lives ranging from days to months depending on reaction conditions.^4,22^ In addition, VX hydrolysis can produce EA-2192, a highly toxic byproduct, when P–O cleavage competes with the desired P–S cleavage pathway. From a decontamination perspective, reaction selectivity and residual toxicity must therefore be considered together with degradation rate.^24^

A-series agents, commonly referred to as Novichok agents, are a newer class of NAs distinct from G- and V-series agents, with A-230, A-232, and A-234 commonly cited as representative liquid agents.^36–39^ These agents are generally described as having phosphoramidofluoridate or phosphonamidic fluoride structures and show low vapor pressures, limited aqueous solubility, and relatively high densities. Because of these properties, A-series agents are less prone to rapid vapor-phase dispersion than G-series agents and are more likely to persist on surfaces, increasing the importance of contact-based exposure.^34,35^ In particular, A-234 has been reported to undergo slow hydrolysis and exhibit high environmental persistence under neutral to mildly basic aqueous conditions. This behavior makes A-234 a relevant case for considering both water availability and surface behavior in practical decontamination environments.^37^ However, publicly available physicochemical and decontamination data for A-series agents remain limited compared with those for G- and V-series agents. More systematic studies are therefore needed to clarify their hydrolysis behavior, persistence, and product distribution in aqueous and surface environments.^34,36,37^ Given these limitations, this review discusses A-series agents only in the context of nerve-agent classification and the current gap in publicly available data. Subsequent quantitative performance comparisons focus on G- and V-series agents and hydrolysis-relevant simulants, for which more extensive data are available.

### 2.2. Simulants

Under the CWC regime, Schedule 1 chemicals are permitted only for research, medical, pharmaceutical, or protective purposes, and their type and quantity are strictly limited; they must also be handled in authorized facilities.^40^ As of March 2026, the OPCW reported 29 designated laboratories in 24 States Parties for the analysis of authentic environmental samples.^41^ As of August 2025, it reported 19 designated laboratories in 14 States Parties for the analysis of authentic biomedical samples.^42^ Because of these toxicity and regulatory constraints, actual NAs are difficult to handle directly in research laboratories.^43^ As a result, experimental studies widely use simulants that have structural similarity to NAs but pose lower handling risks and regulatory burdens.^32^

However, simulant selection should not be based solely on molecular structural similarity. It should also reflect the research objective, such as adsorption, hydrolysis, or oxidation, the target reaction mechanism, and the similarity of degradation products. For studies requiring reproduction of reaction pathways and major degradation products, suitable simulants should mimic the chemical features of actual agents at the reactive phosphorus center, such as the phosphoryl group, and at the leaving group.^32^ Because no single simulant can represent all behaviors of an actual agent, its suitability should be judged by both chemical reactivity and physicochemical properties, including volatility, aqueous solubility, and hydrophobicity.^32,43^ For catalytic hydrolysis studies, dimethyl 4-nitrophenyl phosphate (DMNP), a phosphate ester with a chromophoric *p*-nitrophenyl leaving group, is often used because it allows real-time monitoring of reaction progress.^44^ In vapor-phase adsorption and sensing studies, dimethyl methylphosphonate (DMMP), an organophosphonate with volatility and molecular size similar to those of G-series agents, is widely used as a representative simulant.^45^ However, DMNP and DMMP are purpose-specific probes and do not fully reproduce the reactivity, adsorption behavior, or product distributions of actual nerve agents. The *p*-nitrophenyl leaving group in DMNP facilitates reaction monitoring, but DMNP does not reproduce P–F bond cleavage in G-series agents or the competing P–S and P–O cleavage pathways of VX. Because leaving-group p*K*_a_, molecular electronic structure, and molecular volume influence activation barriers and reaction pathways in organophosphate hydrolysis, simulant suitability should be assessed against the target reaction and study objective rather than structural similarity alone.^46^ Adsorption behavior is also system-dependent: the adsorption properties of DMMP and diethyl chlorophosphate (DCP) have been reported to correlate relatively well with those of GB, whereas DMNP provides a less reliable proxy for GD adsorption.^47^ Thus, simulant–agent correspondence depends on both the material class and the evaluation objective. Simulant-based results should therefore be interpreted as screening evidence, and practical applicability should be established through actual-agent validation and product-selectivity analysis.

## 3. Hydrolysis mechanisms of decontaminants

Detoxification of NAs ultimately involves cleavage of labile bonds at the reactive P center and conversion into less toxic phosphonic acid products. Hydrolysis is one of the most widely studied routes for this conversion, and catalytic hydrolysis represents a central approach.^48^ However, catalytic hydrolysis performance is not governed solely by the intrinsic activity of the catalyst; it is strongly affected by pH, temperature, water availability, and medium composition, such as the buffer system.^23,49^ Therefore, the same catalyst can show different reaction rates and product distributions in bulk aqueous solutions, wet surfaces, or moisture-limited solid interfaces.

For example, the G-series agent GB generally hydrolyzes faster under basic conditions than under acidic or neutral conditions. In this reaction, a nucleophile attacks the P center and promotes P–F bond cleavage. The initial product, isopropyl methylphosphonic acid (IMPA), can undergo further hydrolysis to form methylphosphonic acid (MPA).^28^Eqn (1a) and (1b) schematically show this stepwise hydrolysis pathway of GB.

1a GB + H_2_O → IMPA + HF

1b IMPA + H_2_O → MPA + i-PrOH

By contrast, VX requires consideration of both reaction rate and reaction selectivity. Its hydrolysis can proceed through competing P–S and P–O bond-cleavage pathways, and the reaction conditions determine whether detoxification proceeds or the highly toxic byproduct EA-2192 is formed.^5,22^ Thus, VX decontamination cannot be evaluated solely by degradation rate. Selective promotion of P–S bond cleavage, together with suppression of EA-2192 formation, is essential. Eqn (2a) and (2b) schematically show the representative competing pathways of VX hydrolysis.

2a VX + H_2_O → EA − 2192 + EtOH

2b VX + H_2_O → EMPA + DESH

These differences show that catalytic platforms cannot be assessed by reaction rate alone. Moon *et al.* reported that Zr-based Lewis acid catalysts exhibited high activity for DMNP hydrolysis.^44^ Bandosz *et al.* further demonstrated that solid-surface chemistry and the reaction environment can shift product distribution and effective decontamination behavior.^50^ Accordingly, actual decontamination performance depends not only on catalytic active sites but also on water and base availability, product selectivity, and product-induced catalyst deactivation. Rapid P-centered hydrolysis of DMNP and related *p*-nitrophenyl phosphate simulants should therefore be interpreted as screening evidence; for actual agents such as VX, the competing P–S and P–O cleavage pathways and EA-2192 formation must be evaluated separately.^24,46^ This section summarizes the major catalytic hydrolysis platforms, focusing on the mechanistic factors that govern reaction rate and selectivity in each platform.

### 3.1. Metal oxides

In 1985, Templeton *et al.* elucidated the adsorption and decomposition behavior of DMMP on Al_2_O_3_ surfaces.^51^ Since then, research on metal oxide-based decontamination of organophosphorus compounds has expanded to various oxide compositions. As organophosphorus simulants such as DMMP became practical probes for tracking oxide-surface reactions, understanding has gradually advanced regarding reaction pathways on oxide surfaces and the roles of surface active sites.^52^

**Fig. 4:**
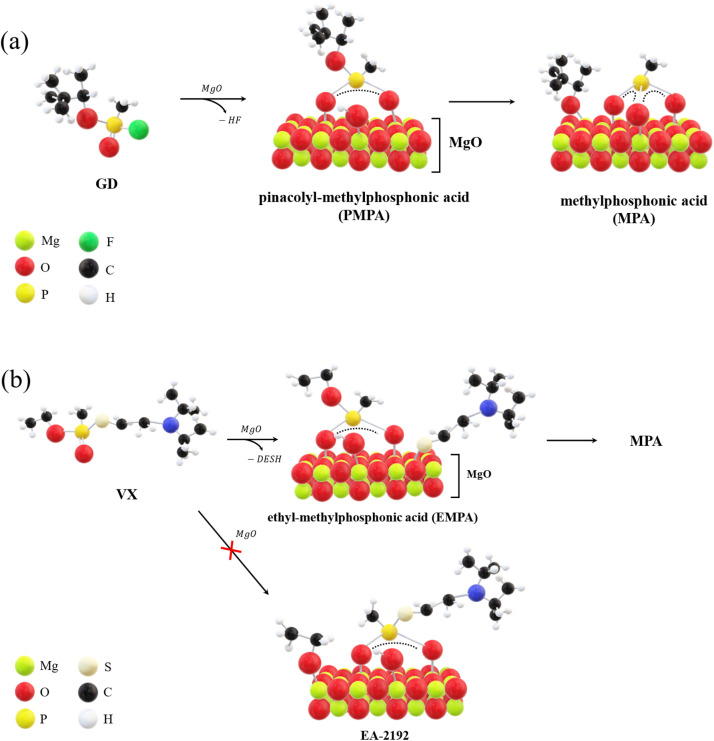
Schematic illustration of the proposed surface-mediated hydrolysis pathways of (a) GD and (b) VX on MgO surfaces.

The decontamination mechanism of metal oxides arises from their bifunctional surface chemistry. Coordinatively unsaturated metal sites (CUSs) act as Lewis acid sites, coordinating the phosphoryl oxygen of the P=O group and activating the P center. Adjacent surface hydroxyl groups or oxide oxygen species then act as nucleophiles and promote bond cleavage.^53,54^Fig. 4 schematically illustrates representative reaction pathways on MgO, in which Lewis-acid activation is coupled with nucleophilic attack by surface O/OH species.

As discussed in Section 2, G-series fluorophosphonate agents such as GD contain a P=O group and a labile P–F bond. They are therefore generally considered to adsorb on metal oxide surfaces through coordination of the phosphoryl oxygen, after which surface hydroxyl groups nucleophilically attack the P center and initiate P–F bond cleavage.^52^ MgO nanoparticles contain defects and low-coordinated sites that create reactive surfaces. In nanocrystalline MgO, the high fraction of exposed surface sites can promote reactive adsorption, where adsorption and decomposition proceed concurrently.^55^

Accordingly, GD undergoes surface-mediated hydrolysis to form PMPA and MPA, with HF generated as a byproduct. In this stepwise mechanism, nucleophilic substitution occurs at the P center of surface-adsorbed GD, leading first to P–F bond cleavage and PMPA formation. PMPA is then converted to MPA through subsequent dealkylation.^16^

By contrast, VX contains a P–S bond, and its hydrolysis can proceed through competing P–S and P–O bond-cleavage pathways. On MgO surfaces, however, ethyl methylphosphonic acid (EMPA) and MPA were reported as the main hydrolysis products.^55^ Notably, EA-2192, a highly toxic byproduct commonly formed during solution-phase alkaline hydrolysis, was not observed on MgO surfaces. This result suggests that, under these surface-catalyzed conditions, P–S bond cleavage was thermodynamically or kinetically favored over the P–O cleavage pathway.

This agent-based mechanistic concept can also be extended to DMMP, a commonly used simulant. In DMMP, methoxy groups (–OCH_3_), rather than fluoride, act as leaving groups. On metal oxide surfaces, these methoxy groups are cleaved stepwise and protonated, releasing methanol. The reaction therefore typically converges to strongly surface-bound methylphosphonate (MP) species, which represent less hazardous decomposition products of the simulant.^52,53^ This pathway is typically observed on nonreducible oxides such as Al_2_O_3_ and MgO, where the P–C bond, specifically the P–CH_3_ bond, tends to remain intact. By contrast, on reducible oxides such as FeO_*x*_ or FeO_*x*_-containing surfaces, oxidative pathways associated with Fe(II)/Fe(III) redox cycling can promote P–C bond cleavage. Thus, the redox activity of the oxide is an important factor governing the extent of decomposition.^53,56^

Metal oxides have been applied to practical protective filters and impregnated protective materials because they are thermally and chemically stable and can be readily shaped.^57^ However, they have several inherent limitations. A major limitation is product poisoning, in which phosphate or phosphonate products strongly bind to surface active sites and rapidly suppress catalytic activity.^58^ Thus, many metal oxide systems behave closer to reactive adsorbents than to catalysts with sustained turnover, with their overall reaction capacity governed by product accumulation and diffusion limitations. As shown for TiO_2_ and ZnO, reactivity also depends strongly on moisture content, surface vacancies, particle size, and surface hydroxylation.^52,57,59^ Accordingly, evaluations of metal oxide-based decontaminants should consider not only initial reactivity but also surface properties, moisture availability, product-induced poisoning, and mass-transfer limitations. Before these materials can be deployed in practical filters, impregnated media, or coatings, studies must also evaluate their residual reactive capacity after repeated exposure, the regenerability of surface active sites, diffusion resistance within shaped bodies, pressure drop, and mechanical durability.

### 3.2. Zeolites and porous silicates

Zeolites are crystalline aluminosilicates represented by the general formula M_*x*/*n*_[(AlO_2_)_*x*_(SiO_2_)_*y*_]·*w*H_2_O. Their frameworks consist of corner-sharing [SiO_4_]^4−^ and [AlO_4_]^5−^ tetrahedra connected through bridging oxygen atoms, forming a three-dimensional negatively charged lattice. Because of this framework structure, zeolites can provide both selective adsorption and ion exchange. They have therefore been studied not only as physical adsorbents but also as reactive sorbents that combine adsorption with chemical decomposition.^60,61^ In particular, conventional physical adsorbents carry the risk of agent re-release and secondary contamination, which has increased interest in reactive sorbents that promote chemical degradation during adsorption. Zeolites are attractive in this context because they combine high specific surface area with ion-exchange-driven catalytic reactivity. Their ability to host and slowly release functional cations, such as Ag^+^, has drawn particular attention.^61^

Initial studies into zeolite-based decontamination examined the degradation of VX and DEPPT on NaY and AgY zeolites.^61,62^ Later mechanistic studies focused on the X-type faujasite NaX because its lower Si/Al ratio than NaY gives the framework a higher negative charge density and increases the density of extra-framework Na^+^ ions in the supercages. These structural features can enhance the nucleophilicity of framework oxygen atoms and provide a favorable environment for leaving-group stabilization. As a result, NaX can promote nucleophilic substitution of organophosphorus compounds. Using solid-state NMR with DMMP, Yang *et al.* showed that framework oxygen atoms inside NaX supercages directly participate in the reaction and drive nucleophilic substitution.^62^ Under low-moisture conditions, framework-bound species and methylphosphonate ions were formed. This finding suggests that zeolite reactivity does not arise from pore adsorption alone. Instead, it originates from the confined supercage environment, where framework oxygen atoms and extra-framework cations act together. Because framework oxygen atoms, extra-framework cations, and pore hydration jointly govern zeolite-based decontamination, their coupled effects must be considered when clarifying structure–reactivity relationships. Accordingly, Table 3 reorganizes reported zeolite-based systems by their major controlling factors and compares changes in half-life, hydrolysis fraction, or removal efficiency across reaction conditions.

**Table 3:** Effects of zeolite-specific factors on nerve-agent and simulant degradation

Key factor	Condition	Reported values	Observed effect	Ref.
Cation exchange	NaY-VX	*t*_1/2_ = 7200 min	AgY shortened *t*_1/2_ by 18.2-fold	61
	AgY-VX	*t*_1/2_ = 396 min	*t*_1/2_ = 396 min	
Substrate identify	AgY-VX	*t*_1/2_ = 396 min	6.2-Fold longer *t*_1/2_ for DEPPT	
	AgY-DEPPT	*t*_1/2_ = 2460 min		
Confined NaX reactivity	NaX-DMMP	*t*_1/2_ = 2.98 min in low-water CCl_4_	Rapid conversion under low-water NaX confinement	62
Water-induced pathway shift	NaX-DFP, dry	Hydrolysis fraction = 6.5%	Water increased the hydrolysis fraction by 6.8-fold	63
	NaX-DFP, water-added	Hydrolysis fraction = 44.5%		
Assisted aqueous removal	Ag-Z micromotor–DCP	∼90% removal in 6 min with 5% H_2_O_2_ + 1% SDS	Oxidant/surfactant-assisted removal; not a hydrolysis-only benchmark	60

As Table 3 shows, zeolite-mediated degradation reflects the combined effects of cation composition, substrate identity, the confined pore environment, and hydration state rather than zeolite type alone. Taken together, the H_2_^18^O-labeling and DFP studies show that water is not merely a solvent but a mechanistic variable that influences the preferred site of nucleophilic attack and the balance between elimination and hydrolysis.^63,64^ Water can therefore promote subsequent hydrolysis and active-site regeneration; when present in excess, however, it can solvate extra-framework Na^+^ ions and interact strongly with framework oxygen atoms, thereby lowering nucleophilicity or restricting substrate access. This water dependence is revisited in Section 4.

Ordered mesoporous silicas (OMSs) are important porous silicate platforms for the capture and decontamination of OPCs. Their silica frameworks are chemically and thermally stable and contain abundant surface silanol (Si–OH) and siloxane (Si–O–Si) groups. These surface groups can participate in adsorption and surface reactions. More importantly, the ordered mesoporous structure allows the pore size, diffusion pathway, and molecular accessibility to be adjusted. This structural tunability became clear after MCM-41 established the concept of mesoporous silica frameworks.^65^ It was further expanded by the development of SBA-15, which has larger pores and thicker pore walls. As a result, porous silicate platforms could be designed by considering diffusion rate, residence time, and functional-group incorporation.^66^

**Fig. 5:**
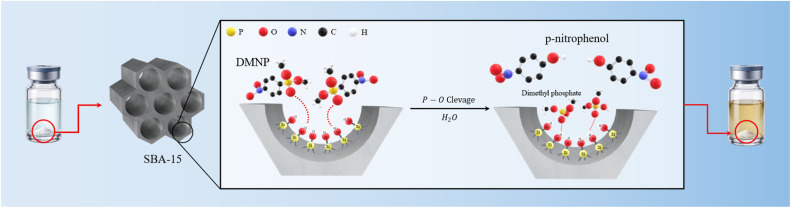
Schematic illustration of DMNP adsorption and hydrolysis within SBA-15 mesopores.

On silica surfaces, OPC adsorption is mainly driven by hydrogen bonding between the phosphoryl oxygen of the P=O group and surface silanol groups. This interaction has been confirmed by IR spectroscopy and TPD analysis.^67^Fig. 5 schematically shows DMNP adsorption on SBA-15 and its subsequent hydrolysis pathway.

However, unlike typical metal oxides (*e.g.*, TiO_2_ and ZrO_2_), silica lacks strong surface Lewis acid sites. It therefore has limited ability to promote hydrolysis directly through P=O bond polarization. Nevertheless, surface silanol/silanolate species (Si–OH/ SiO^−^) and the pore hydration layer can still influence reaction kinetics by supplying nucleophiles and stabilizing ionic intermediates and products. Bahia *et al.* reported that bare silica materials, including MCM-41, SBA-15, and silica gel, catalyzed the hydrolysis of the model phosphate ester BDNPP.^68^ They attributed this activity to acid–base cooperation between silanol and siloxide sites and to the surface hydration state, which affected both the reaction rate and induction period. These results indicate that silica can participate in hydrolysis rather than serve only as an inert support.

Therefore, decontamination strategies based on silica have generally evolved from using the intrinsic surface properties of silica alone toward using silica as a high-surface-area support for loading or integrating more reactive species. Candel *et al.* introduced base catalysts such as K_2_CO_3_ into mesoporous silica nanoparticles and increased the hydrolysis conversion of DCNP to approximately 95%.^69^ They attributed this enhancement to deprotonated surface silanolate species (Si–O^−^), which acted as nucleophiles and attacked the P center. Rotter *et al.* incorporated MCM-41 containing highly dispersed TiO_2_ nanoparticles into a PDMS matrix to create a reactive barrier against GB and VX.^70^ In this system, the mesoporous framework slowed agent diffusion and prolonged contact with the active sites. Overall, silica materials can serve as multifunctional decontamination platforms rather than simple physical adsorbents: pore-structure control can optimize mass transport, while surface functionalization can impart catalytic activity. For practical deployment as pellets, membranes, PDMS barriers, or coated fibers, evaluations must extend beyond powder-form or model-suspension reactivity to include pore accessibility after shaping, retention of reactivity under varying humidity, leaching of active species, and durability over repeated use.^63,64,70^

### 3.3. Carbon-based materials & composites

Porous carbon materials based on activated carbon (AC) have long served as standard materials in respirator canisters and adsorbent beds because of their high specific surface area and well-developed microporosity. Early AC studies mainly focused on physical adsorption kinetics and breakthrough prediction using models such as the Wheeler equation.^71^ However, physical adsorption alone cannot fully prevent reversible agent desorption and secondary contamination. Recent efforts have therefore moved toward impregnated AC and carbon-based composites that combine adsorption with chemical degradation. In these materials, carbon pores concentrate agents and provide diffusion pathways, whereas incorporated metal oxides, metal salts, or basic functional groups drive chemical decomposition.

In carbon-based decontamination systems, the carbon framework mainly captures agent molecules and delivers them to active sites. Although oxygen-containing groups on pristine AC can support limited surface reactions, effective decomposition is largely governed by supported active species. Carbon pores act as an adsorptive reservoir and diffusion network, whereas loaded metals, oxides, or basic functional groups provide active sites for chemical bond cleavage.^72^ Because excessive loading can block micropores, reduce specific surface area, and increase diffusion resistance, these systems must balance reactivity with adsorption capacity.^72^

**Fig. 6:**
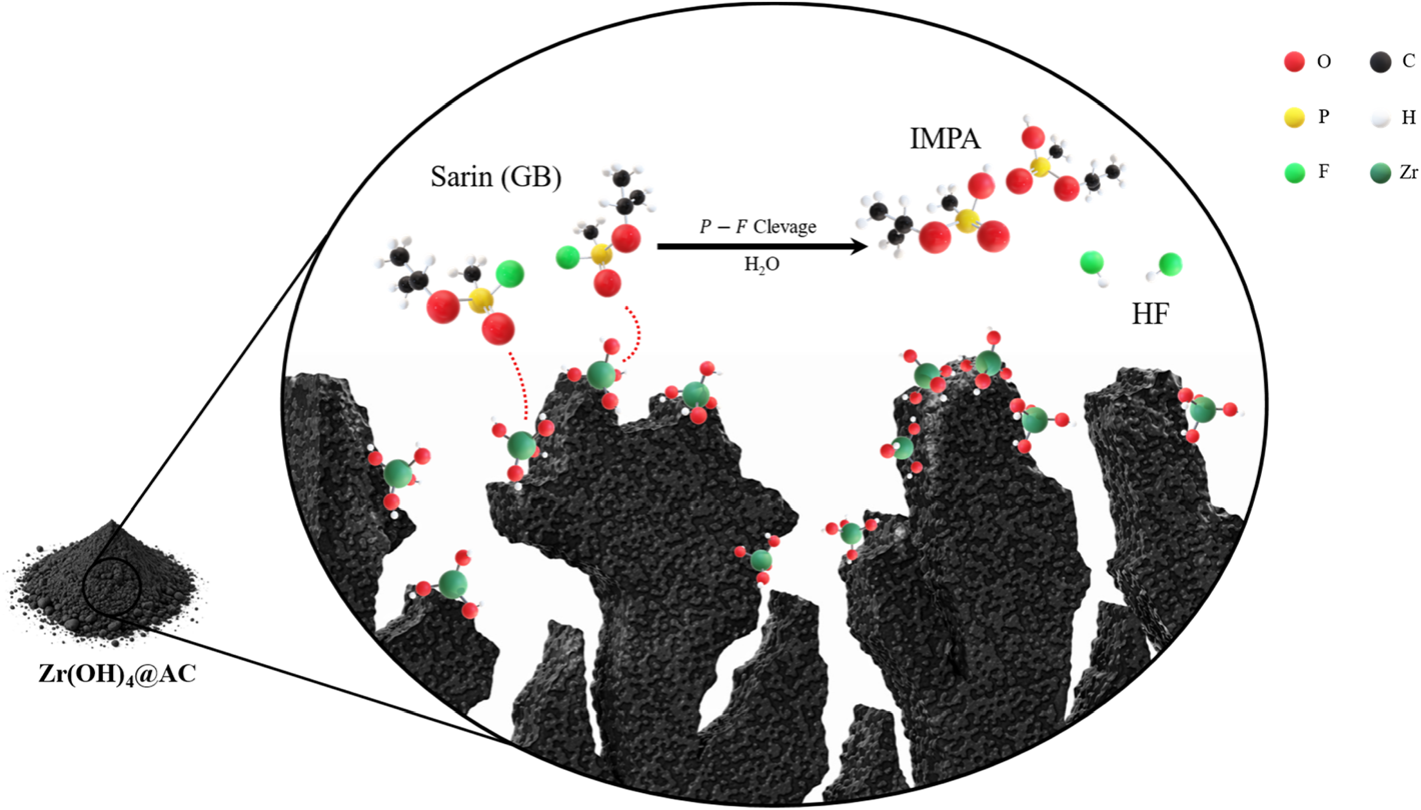
Schematic illustration of coupled GB adsorption and hydrolysis at Zr(OH)_4_ active sites within AC pores.

This concept becomes more evident in hybrid architectures. Imran *et al.* developed an *in situ* decontamination system using a Zr(OH)_4_@W-ACF composite, in which Zr(OH)_4_ was grown on the surface of woven activated carbon fabric (W-ACF). This design combined the rapid adsorption capacity of W-ACF with the high hydrolytic activity of Zr(OH)_4_.^73^ In this system, the carbon support dispersed the agent molecules over a large surface area and increased their probability of contact with active sites. The composite showed degradation behavior consistent with first-order kinetics. Fig. 6 schematically illustrates this coupled adsorption-hydrolysis mechanism: GB is first adsorbed on Zr(OH)_4_ active sites supported inside AC pores, followed by coordination of the P=O group and subsequent P–F bond cleavage to form IMPA and HF. By contrast, Xie *et al.* proposed a microfibrous entrapped activated carbon (MEAC) structure that enhanced the dynamic adsorption performance of DMMP and prolonged its breakthrough time.^74^ In this case, the carbon architecture mainly improves mass transport and adsorption efficiency rather than directly promoting hydrolysis.

In carbon-based reaction systems, deactivation caused by product accumulation and the feasibility of post-adsorption treatment are important design variables. During prolonged operation or under high-temperature conditions, phosphate and phosphonate species can accumulate inside carbon pores, poison active sites, and reduce reactivity.^75^ To address this issue, Osovsky *et al.* proposed treating agent-loaded carbon with an H_2_O_2_ solution after adsorption.^76^ In this strategy, agents concentrated within the carbon pores undergo H_2_O_2_-assisted perhydrolysis, which can suppress the formation of highly toxic byproducts and facilitate adsorbent regeneration. Because H_2_O_2_ changes the reaction pathway, this process should be interpreted as an oxidant-assisted post-treatment rather than as hydrolysis catalyzed directly by the carbon framework. Overall, carbon-based decontaminants are best viewed as platforms that integrate adsorption, concentration, reaction, and regeneration. Their practical performance should therefore be evaluated in terms of pore-scale mass transfer, the identity of supported active species, product-induced deactivation, and post-treatment feasibility. For applications in filters and packed beds, breakthrough capacity, pressure drop, performance under humid-air conditions, and spent-carbon handling are key engineering metrics.^71–76^

### 3.4. Metal–organic frameworks (MOFs)

MOFs are crystalline porous materials formed by the periodic assembly of metal nodes, or secondary building units (SBUs), and organic linkers. They have attracted attention as promising decontamination catalysts because adsorption, activation, and hydrolysis can occur within a single platform. Among them, Zr(iv)-carboxylate MOFs have become a major focus because they combine high chemical and thermal stability with strong node-centered Lewis acidity. Mondloch *et al.* reported that NU-1000, which contains Zr_6_ nodes, showed high catalytic activity for the hydrolysis of organophosphorus bonds.^77^ Moon *et al.* further showed that MOF-808, a 6-connected Zr-MOF with many exposed or exchangeable Zr–OH/H_2_O ligands, achieved sub-minute half-lives for DMNP hydrolysis.^44^ This result indicates that node structure and active-site exposure strongly control reactivity. Recent studies on defect control and single-atom modification have also confirmed that exposed Zr active sites and their local coordination environment are key variables governing hydrolytic activity.^78,79^

OP agent hydrolysis by Zr-MOFs is generally described as a sequence of Lewis acid activation, nucleophilic attack, product release, and active-site regeneration. First, the phosphoryl oxygen of the P=O group coordinates to a Lewis-acidic Zr(iv) center. This coordination polarizes the P=O bond and increases the electrophilicity of the P center.^23,77^ Zr-bound aqua or hydroxo ligands, or water molecules activated near the node, then act as nucleophiles and attack the P center. The reaction is proposed to proceed through a pentacoordinate intermediate, followed by cleavage of a labile bond such as the P–F bond in GB or the P–S bond in VX.^23^ However, this process is not governed simply by the presence of water. Its rate and pathway depend strongly on the exchangeability of Zr-bound water molecules, the proton topology of the node, and the availability of a basic environment that can neutralize acidic byproducts formed during the reaction.^77^ More recently, Liao *et al.* combined p*K*_a_ measurements with kinetic analysis of NU-1000 to refine active-site assignments in Zr-MOFs.^80^ Their results indicate that µ-hydroxo ligands can convert to substitution-inert µ-oxo bridges under alkaline conditions, suggesting that Zr-MOF reactivity cannot be explained solely by assigning Zr(iv)–OH–Zr(iv) bridging hydroxo groups as the principal active sites. Rate control also depends on framework structure. In UiO-66, substrate binding through displacement of node-bound water was interpreted as rate-determining, whereas in NU-1000, solution-phase hydroxide attack on MOF-activated DMNP was proposed to limit the rate.^80^ Accordingly, active-site functionality in Zr-MOFs should not be defined by metal-node identity or defect density alone. It should instead be interpreted through a coupled set of molecular and transport descriptors: node protonation state, the abundance of Zr-bound aqua sites (Zr–OH_2_), substrate-binding equilibria, pore transport, solution pH, and base availability.

In this process, water acts as both an essential component and a potential inhibitor. Momeni *et al.* showed through computational studies of GB hydrolysis that water is required as a nucleophile source, whereas excess water can competitively coordinate to Zr active sites and block agent access.^23^ By contrast, partial dehydration through thermal treatment or defect introduction can create CUSs and reduce this coordination barrier, thereby accelerating the initial reaction.^77,81^ Therefore, MOF catalyst design should account for the local form and distribution of water inside the pores.

The catalytic performance of MOFs also depends strongly on active-site accessibility, which is controlled by node connectivity and pore structure. Structures with large mesoporous channels, such as NU-1000, expose SBUs along the internal channels and facilitate substrate diffusion. Low-connectivity structures, such as MOF-808, provide a high density of accessible active sites per node and therefore show high reactivity.^44,82^ By contrast, UiO-66-type MOFs have high stability, but their 12-connected nodes and relatively narrow pores can limit the fraction of nodes that participate in the reaction. Defect engineering and functional-group incorporation are therefore important strategies for improving their reactivity. For V-series agents such as VX, chemoselectivity must also be considered along with reaction rate. In amine-functionalized UiO-67 derivatives such as UiO-67-N(Me)_2_, P–S bond cleavage was reported to predominate over P–O cleavage, the pathway that forms EA-2192.^83^

Finally, moving MOF catalysts from powder form into practical protective systems requires suitable shaping methods and interfacial stability. To meet this requirement, several approaches have been explored, including polymer nanofiber-based MOFabric,^84^ textile-grown MOF-cloth,^85^ and hydrogel composites designed to provide water and buffer species.^81^ However, even after shaping, key bottlenecks remain for commercialization. These include binder-induced pore blockage, active-site poisoning, product accumulation, and the need to maintain water and base supply under dry operational conditions.^82^ Accordingly, next-generation MOF decontamination technologies should move beyond the synthesis of new frameworks toward integrated designs that preserve active-site accessibility and local water/base microenvironments in shaped forms. For practical formats such as protective textiles, coatings, and hydrogel composites, evaluations should include MOF-loading uniformity, stable adhesion to fibers or binders, resistance to particle loss during repeated bending, abrasion, and washing, retention of reactivity after humid–dry cycling, and active-site regenerability after exposure to agents and their degradation products.^81,84–86^

### 3.5. Polyoxometalates (POMs)

Whereas MOFs discussed in Section 3.4 are extended three-dimensional porous lattices built from metal nodes and organic linkers, POMs are discrete molecular metal–oxo anion clusters. Unlike MOFs, whose reaction rates can be affected by diffusion into internal pores, POMs show relatively small internal diffusion limitations when used as molecular catalysts in solution or as nanoscale units with exposed external surfaces. Their inorganic metal–oxo skeletons contain no organic linkers, allowing atomic-level control over structure and composition. POMs have therefore been regarded as molecular models of metal oxides and as platforms that connect homogeneous and heterogeneous catalysis.^87,88^

In OPNA decontamination, POM-based studies have followed two main directions. One strategy uses lacunary POMs as ligands to incorporate strongly Lewis-acidic metal centers such as Zr(IV), Ti(IV), and Ce(IV), thereby forming molecular Lewis acid catalysts. The other uses the basic oxygen sites of polyoxoniobates for general base catalysis. These strategies are significant because they enable more direct analysis of active-site structures and reaction pathways than is typically achievable in porous solids such as MOFs or AC.^87,88^

The hydrolysis mechanism of Zr-substituted POMs is based on Lewis acid activation. Coordination of the phosphoryl oxygen of the P̄O group to a Zr(IV) center polarizes the P center. A nearby Zr–OH group or a nucleophile generated from coordinated water then attacks the P center, leading to a pentacoordinate intermediate and subsequent leaving-group departure.^49^ In some Zr-polytungstate systems, the catalyst can undergo dimer-to-monomer conversion in response to solution equilibrium or agent exposure, rather than remaining as a static single structure. This conversion exposes open Zr sites and has been linked to enhanced reactivity. Compared with Zr-MOFs with multiple binding sites, this single-site character can reduce product binding energy and help suppress catalyst poisoning caused by product accumulation.^89^

By contrast, polyoxoniobates are driven by the basicity of the oxo framework rather than by Lewis acidic metal centers. Studies on GB and DMMP degradation have shown that basic oxo sites in POMs follow a general base catalysis pathway. These sites can dissociate water molecules and assist hydroxide attack on the P center. However, turnover can become limited as the reaction proceeds because methylphosphonic acid-type products bind strongly to the polyanion or because POM oxygen sites become protonated.^90,91^ Thus, although the strong basicity of polyoxoniobates promotes initial hydrolysis, product binding and structural disorder can limit sustained catalytic turnover.

A key variable in POM catalysis is sensitivity to the reaction medium. In solution-phase reactions, buffers do more than control pH. They can also act as chemical species that directly participate in the reaction. For example, acetate can accelerate hydrolysis by forming hydrogen bonds with Zr-bound ligands or by acting as a local base. In contrast, phosphate buffers or the reaction product MPA can directly coordinate to Zr active sites and inhibit the catalyst.^49,92^ Therefore, the structural tunability of POMs is accompanied by sensitivity to solution speciation and competitive coordination. Thus, practical decontamination performance should be interpreted by separating the effects of buffer type and concentration from product-induced self-inhibition.

Finally, immobilization has been actively investigated to overcome the limitations of solution-phase POM catalysts and improve field applicability. This strategy often exploits the anionic character of POMs, enabling their electrostatic immobilization on positively charged silica nanoparticles or within polymer matrices.^93^ Gel-network composites have also been proposed to capture and degrade organic agents simultaneously.^88^ Overall, the well-defined active-site structures of POMs facilitate mechanistic interpretation, while immobilization and composite formation provide routes toward practical deployment.^93^ However, activity measured in solution cannot be assumed to remain unchanged after immobilization or incorporation into a composite. Evaluations should therefore address POM leaching, diffusion limitations within the support, matrix swelling, ion exchange with solution-phase species, and stability over repeated use. Future studies should report not only solution-phase rates but also turnover under immobilized conditions and performance across regeneration cycles.^88,93^

## 4. Field-relevant decontaminant selection

This section synthesizes the engineering variables required to interpret laboratory-reported decontamination performance under practical conditions. The discussion is based on the NA classes and physicochemical properties summarized in Section 2 and the hydrolysis mechanisms compared across material classes in Section 3. Real decontamination environments differ from ideal aqueous systems because they involve limited water supply, heterogeneous surfaces, mass-transfer resistance, finite application time, and constraints on recovery or waste treatment. Hydrolysis-based decontaminants should therefore be selected according to the intended water/base regime, contaminated medium, application form, and waste-management capacity.

Specifically, this section discusses:

• The effects of environmental and operational conditions on hydrolysis kinetics, reaction selectivity, and sustained performance.

• The reinterpretation of literature-reported data using water and base availability as classification criteria, together with integrated trends in reactivity.

• Selection criteria for hydrolysis-based decontaminants suited to contaminated-surface treatment, respiratory-protection filters, reactive coatings, protective textiles, spent-sorbent regeneration, and decontamination-effluent management.

### 4.1. Effects of operation conditions on decontamination performance

Hydrolysis-based decontamination performance is commonly reported using half-life (*t*_1/2_), observed rate constant (*k*_obs_), or the time required to reach a defined conversion or removal endpoint. When the original study reports *k*_obs_ from first-order or pseudo-first-order fitting, *t*_1/2_ and *k*_obs_ are related by eqn (3).^83,92^3
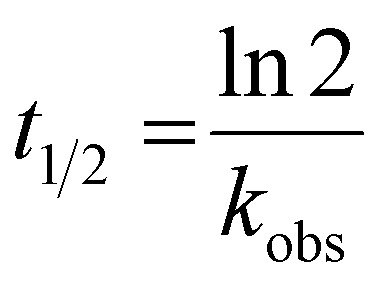


Not all reported half-lives have the same kinetic basis. Some values come from first-order or pseudo-first-order fits. Others are reported as 50% conversion times, 90% removal times, or apparent degradation times in solid-state, interfacial, or sorbent-based tests. These metrics should therefore not be treated as directly comparable intrinsic rate constants. They are apparent performance descriptors that reflect the behavior of each test system.

As discussed in Section 3, apparent decontamination performance depends on both material chemistry and test conditions. Important variables include water supply, buffer or base addition, reaction phase, catalyst loading, agent loading, interfacial mass transfer, contaminant spreading, product accumulation, and active-site poisoning.^49^ Half-lives and rate constants obtained under ideal aqueous conditions must therefore be interpreted within their original experimental context. Practical performance should be evaluated against the constraints of actual decontamination environments.

Table 4 summarizes representative hydrolysis-based decontamination data obtained under different water and base conditions. As shown in Table 4, even the same decontaminant can show different apparent performance as water and base availability change. Mondloch *et al.* reported that NU-1000 gave a half-life of 3 min for GD in an *N*-ethylmorpholine-buffered aqueous solution at pH ≈ 10. Under solid-state conditions at 50% RH without an external base, the half-life increased to 36 min.^77^ Luo *et al.* reported a similar condition dependence for Im@MOF-808. The material showed a half-life below 0.5 min in aqueous solution, whereas the half-life increased to 15 min under humid solid-state conditions at 99% RH.^94^

**Table 4:** Representative examples showing how operational conditions affect apparent hydrolysis kinetics for the same catalysts

Catalytic decontaminant	Agent	Operational conditions		*t*_1/2_ (min)	*k*_obs_ (min^−1^)	Ref.
		Water condition	Base condition			
NU-1000 (Zr-MOF)	GD	Aqueous water + *N*-ethylmorpholine buffers	pH ≈ 10	3	0.231	77
		Solid phase + 50% RH	No external base/buffer added	36	0.0193	
Im@MOF-808 (Zr-MOF + imidazole)	DMNP	Pure water (unbuffered)	No external base/buffer, internal base present	<0.5	>1.39	94
		Solid phase + 99% RH	No external base/buffer, internal base present	15	0.0462	
UiO-66	DMNP	20 mM NEM buffer	pH 8	≈167	0.00415	95
		20 mM NEM buffer	pH 10	≈70	0.00990	
NU-1000 (Zr-MOF)	DMNP	20 mM NEM buffer	pH 8	340	0.00204	
		20 mM NEM buffer	pH 10	108	0.00642	

Aqueous-phase reactions also depend strongly on pH and buffer conditions. Palomba *et al.* examined DMNP hydrolysis over UiO-66 and NU-1000 at a fixed *N*-ethylmorpholine (NEM) buffer concentration of 20 mM.^95^ When the pH increased from 8 to 10, the UiO-66 half-life decreased from approximately 167 to 70 min, and *k*_obs_ increased from 0.00415 to 0.00990 min^−1^. For NU-1000, the half-life decreased from 340 to 108 min, and *k*_obs_ increased from 0.00204 to 0.00642 min^−1^. These trends show the coupled roles of pH and buffer environment. Basic conditions increase the availability of nucleophilic species, while the buffer helps maintain the local reaction environment by neutralizing acidic products.

Rate data organized only by material type can obscure the role of water and base supply. Differences in reported *t*_1/2_ or *k*_obs_ can arise from different test environments rather than from intrinsic material activity alone. A more objective comparison of practical decontamination performance therefore requires classification criteria that separate material identity from the operational water/base environment. The following section develops this classification.

### 4.2. W/B-class mapping of literature-reported decontamination performance

#### 4.2.1. Dataset construction and kinetic normalization

This section defines the criteria used to construct the dataset and normalize kinetic data for comparing literature-reported decontamination performance across W/B-Class. The dataset covers the material classes discussed in this review: metal oxides, zeolites and porous silicates, carbon-based materials, MOFs, and POMs. As noted in Section 2.1, publicly available data for A-series agents remain insufficient with respect to hydrolysis kinetics, product distributions, and test conditions that permit comparison across W/B-Class. Accordingly, the quantitative W/B-Class dataset focuses on actual G- and V-series nerve agents and hydrolysis-relevant simulants and excludes A-series agents from quantitative mapping.

The quantitative dataset included entries with kinetic metrics that could be expressed as an apparent *t*_90_, including *t*_1/2_, *k*_obs_, and directly reported 90% removal or degradation times. Each entry also had to provide sufficient information to identify the corresponding water and base/buffer conditions. Table S1 lists the W/B-Class assignment, the kinetic metric reported in the original study, the apparent *t*_90_, and the *t*_90_ bin used to define marker size in Fig. 8. The 90% removal or degradation endpoint has been used as a practical metric for comparing CWA decomposition efficiency, but in this review it is treated only as an apparent system-level endpoint rather than as an intrinsic kinetic constant.^96^

Directly reported 90% removal or degradation times were retained as the apparent *t*_90_. In this review, *k*_app_ is defined as a first-order-equivalent descriptor used to convert the kinetic metric reported in each source into an apparent *t*_90_ and, where possible, a loading-adjusted apparent descriptor. When *k*_obs_ was obtained from first- or pseudo-first-order fitting, we set *k*_app_ = *k*_obs_. When only *t*_1/2_ was reported, we calculated *k*_app_ = ln2/*t*_1/2_. When *t*_90_ was reported directly, we calculated *k*_app_ = ln10/*t*_90_. Half-lives or endpoints reported without an explicit kinetic model were treated only as first-order-equivalent system descriptors rather than intrinsic rate constants. Eqn (4) gives the corresponding apparent *t*_90_.4
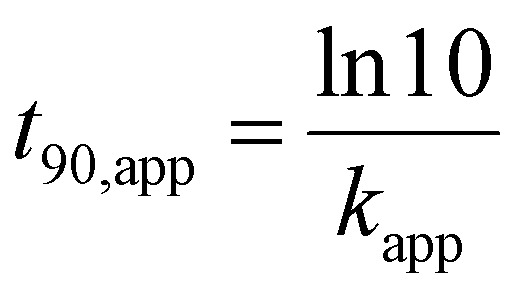


Because the source studies differ in decontaminant loading, agent loading, reaction phase, and water/base conditions, the apparent *t*_90_ values in Table S1 should not be interpreted as an absolute ranking of intrinsic catalytic activity. To address this limitation, Table S2 summarizes the experimental format, reaction medium, loading information used for normalization, *k*_app_, and, where calculable, a loading-adjusted apparent descriptor. For bulk-aqueous or slurry systems, we used *k*_app_*V*_rxn_/*m*_decon_. For dry, humid, or droplet-on-solid systems without a defined bulk-liquid volume, we used *r*_app,m_ = *k*_app_*n*_agent_/*m*_decon_. When the source did not report the information required for calculation, we assigned NC (Not Calculated) rather than estimating missing values. These two loading-adjusted descriptors were used only as format-specific supplementary metrics. Because they differ in units and physical meaning, we did not compare them directly: k_app_*V*_rxn_/*m*_decon_ was interpreted only within bulk-aqueous or slurry systems, whereas *r*_app,m_ was interpreted only within dry, humid, or interfacial systems. We did not use BET surface area as a common normalization basis because it was not reported consistently and does not directly represent the number of accessible reactive sites in shaped bodies or under conditions involving hydration layers, pore occlusion, or interfacial water.

For VX, the formation of the highly toxic byproduct EA-2192 is a key performance criterion alongside degradation rate. Table S2 therefore records the EA-2192 status of each VX entry as Detected, Not detected under the reported analytical conditions, or Not assessed when the source did not evaluate or report the relevant outcome. Because many source studies did not consistently report replicate measurements, standard deviations, confidence intervals, or fitting errors, we did not impute unreported statistical uncertainties. Instead, Tables S1 and S2 identify the kinetic basis available from each source and whether the corresponding descriptors could be calculated.

#### 4.2.2. Operational definition of W-Class and B-Class

**Fig. 7:**
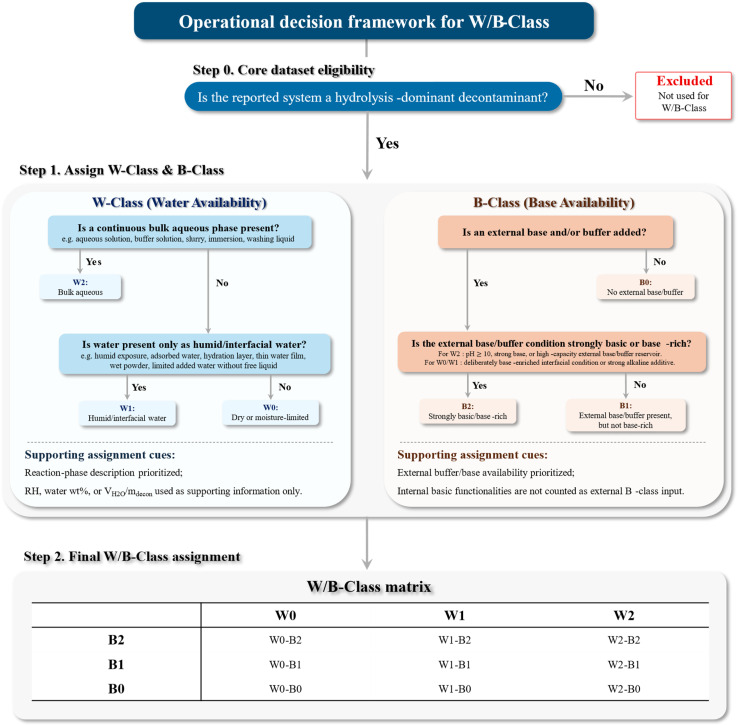
Operational decision framework for W/B-Class assignment. Hydrolysis-dominant entries were first screened, and non-core cases were excluded before W/B assignment. W-Class was assigned from interfacial water availability, whereas B-Class was assigned from external base/buffer availability using reported phase, RH, water loading, pH, and buffer/base information.

This section reclassifies literature-reported decontamination test conditions using two operational variables: water availability (W-Class) and external base/buffer availability (B-Class). The W/B-Class criteria compare the operational environment in which hydrolysis occurred. W-Class describes the physical form of water at the decontaminant–agent interface. B-Class describes the external base or buffer supplied to the reaction medium. Fig. 7 shows the decision flow used to assign literature conditions to W-Class and B-Class.

**Fig. 8:**
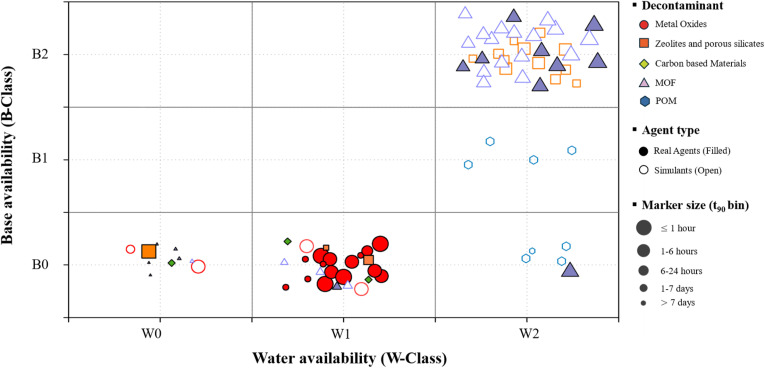
W-Class/B-Class map of core hydrolysis-dominant decontamination entries. Data points correspond to the core entries listed in Table S1 and were assigned according to the criteria defined in Fig. 7 and Section 4.2.2. Marker shape indicates decontaminant class, marker filling distinguishes authentic nerve agents from simulants, and marker size represents binned apparent *t*_90_. Apparent *t*_90_ values are system-level descriptors derived from the original kinetic metrics and should not be interpreted as intrinsic catalytic rate constants. Small offsets within each W/B cell were used only to reduce marker overlap. Loading information and loading-adjusted apparent descriptors, where calculable, are provided in Table S2, together with the EA-2192 assessment status for all VX entries. The loading-adjusted descriptors in Table S2 are interpreted only within the same reaction format and were not used as cross-regime intrinsic activity rankings.

##### 4.2.2.1 Water availability, W-class

W-Class was assigned according to the water state at the decontaminant–agent interface, not total water content alone. Water availability affects nucleophile supply and active-site accessibility in Zr-MOFs.^23^ It can also change the reaction pathway and rate in zeolites.^63,64^ In metal oxide systems, adsorbed water and surface hydroxyls play important roles in hydrolysis activity.^97,98^ Accordingly, we used the reaction-phase description reported in each source as the primary criterion and RH, water content (wt%), *V*_H_2_O_/*m*_decon_, and, where available, pore-filling information as secondary operational criteria. For porous decontaminants, pore-filling information was used only as a secondary operational guide because pore-confined or capillary-condensed water is not equivalent to a freely mixed bulk aqueous phase.^99^

• W0 (dry or moisture-limited): conditions with no or very limited external water supply. This class includes dry conditions, oven-dried conditions, dry-gas conditions, solvent-free agent-on-solid tests, and tests without intentional water or humidity supply. When RH was reported, tests conducted at RH ≤ 10% were assigned to W0. Reactions in this regime rely mainly on surface hydroxyl groups, structural water, or trace adsorbed water. W0 therefore denotes a moisture-limited operational regime in which reactions proceed without intentionally supplied water or a continuous aqueous phase.

• W1 (Humid or interfacial-water): conditions without bulk liquid water but with adsorbed water, hydration layers, pore-confined water, thin water films, or added interfacial water. This class includes controlled-RH conditions, explicitly reported exposure to ambient humidity, prehydrated solids, high-RH solid-state tests, moist solid surfaces, and wet powders. When RH was reported, conditions at RH > 10% were assigned to W1 if the source did not identify a free liquid phase. When the added-water volume was reported, conditions with 0 < *V*_H_2_O_/*m*_decon_ ≤ 1 mL g^−1^ were assigned to W1 if the system was described as a wet powder or an interfacial-water condition. When pore-volume data were available, a water loading at or below the estimated pore-filling level was used as a secondary criterion for W1. High RH or a moist solid surface was not assigned to W2 unless a continuous bulk aqueous phase was present.

• W2 (bulk aqueous): conditions with continuous liquid water. This class includes pure water, D_2_O solutions, aqueous solutions, buffer solutions, alkaline solutions, slurries, washing liquids, and aqueous post-treatment solutions in which the aqueous phase serves as the reaction medium. When reaction volume was reported, *V*_aq_/*m*_decon_ ≥ 10 mL g^−1^ was used as the operational threshold for W2. Conditions explicitly described as solutions, slurries, immersion tests, or washing liquids were assigned to W2 even when the complete reaction volume was not reported. In this regime, bulk pH and dissolved base/buffer conditions can be defined operationally.

These numerical thresholds are operational guides for classifying heterogeneous literature conditions consistently, not strict phase boundaries. For cases with 1 < *V*_H_2_O_/*m*_decon_ < 10 mL g^−1^, or when water loading alone did not clearly distinguish W1 from W2, the reaction-phase description in the original source took precedence. Conditions described as wet powders, moist solid surfaces, thin water films, or interfacial-water systems were assigned to W1 when no free-flowing liquid was present. Conditions described as solutions, slurries, immersion tests, or washing liquids were assigned to W2 when a continuous bulk liquid phase was present. Ambiguous cases that could not be resolved using these criteria were excluded from the core dataset.

##### 4.2.2.2 Base availability, B-class

B-Class was assigned according to the external base or buffer supplied to the reaction medium. We used the presence or absence of an externally added base or buffer as the primary criterion and pH/pD, external base/buffer concentration (*C*_base/buffer_), and base/buffer identity as secondary criteria. Basic functionalities incorporated into the decontaminant, including amine linkers, imidazole groups, BPEIH, and polymeric internal bases, were treated as intrinsic material features and were not counted as external B-Class inputs.^81,83,94^ Bases or buffers used only for post-reaction extraction or analysis were excluded from B-Class assignment. This rule distinguishes operational base/buffer demand from the intrinsic basicity of the material. Accordingly, B0 denotes the absence of an externally added base or buffer, not the absence of intrinsic basic functionality within the decontaminant.

Because W2 environments contain a bulk aqueous phase, pH/pD and the external base/buffer reservoir can serve as direct operational criteria. We therefore applied the same hierarchy directly to W2 conditions: the presence or absence of an externally added base or buffer was the primary criterion, whereas pH/pD, *C*_base/buffer_, and base/buffer identity were secondary operational criteria. For VX, solution conditions can alter both the degradation rate and the selectivity between P–S and P–O bond cleavage, so EA-2192 formation must also be evaluated.^22,24^ Depending on their identity and concentration, buffer species can either accelerate hydrolysis or inhibit active sites.^49,92,100^

• B0: water-only, unbuffered, acidic, or near-neutral aqueous conditions without an external base or buffer. Unbuffered water was assigned to B0 even when its pH was near neutral. Acidic conditions were also included in B0 because they do not provide an effective base reservoir.

• B1: conditions in which an external base or buffer is present, but the medium is neither base-rich nor strongly basic. In W2 systems, neutral to mildly basic buffer/base conditions at pH/pD < 10 were generally assigned to B1. This regime includes conditions in which the added buffer or base supports pH control, proton transfer, or local reaction-environment control, but neither a strongly basic bulk medium nor a high-capacity base reservoir dominates the reaction. For example, pD 7 Tris–HCl buffer conditions were assigned to B1 because they were neither strongly basic nor base-rich, even though Tris can participate directly in the reaction.^92^ Buffer concentration alone therefore did not justify assignment to B2; near-neutral buffer conditions remained in B1 even at concentrations of 0.1 M or higher.

•B2: Base-rich or strongly basic aqueous conditions. In W2 systems, conditions were assigned to B2 when the original source reported pH/pD ≥ 10, strong-base addition, or a high-capacity external base/buffer reservoir. When pH/pD was not reported, base/buffer identity and concentration served as secondary criteria. Conditions supplied with a strong base or a clearly basic external buffer at concentrations of ≥0.1 M were assigned to B2. Conditions such as 0.4–0.5 M NEM at pH ≥ 10 were assigned to B2 because they provide a large external base/buffer reservoir during hydrolysis.^44,77^ The same rule was applied to UiO-67 derivatives and MOF-fiber systems tested in 0.45–0.5 M NEM.^83,86^ B2 does not mean that base-promoted background hydrolysis dominates the reaction. It means that external base/buffer availability is high.

In W0 and W1 environments, bulk-solution pH cannot be applied consistently. Accordingly, B-Class assignment in these regimes was based not on the numerical pH value itself but on whether an external base or buffer was intentionally supplied to the dry or interfacial reaction environment and whether the additive could generate a local alkaline microenvironment or brine-like microphase.

• B0: dry, humid, or interfacial-water conditions without an added external base or buffer. Systems with internal basic functionality were still assigned to B0 when no external base or buffer was supplied.

• B1: W0/W1 conditions with a limited external base or weak buffer. The added base or buffer could influence the local interfacial environment, but the condition was not interpreted in terms of bulk-aqueous pH. When the external base/buffer concentration was reported, limited additions in the range of 1–50 mM were generally used as an operational guide forB1 assignment. However, even within this range, cases explicitly described as involving a strong base, a deliquescent salt/base additive, or the formation of a base-rich microphase were evaluated separately for possible assignment to B2.

• B2: W0/W1 conditions in which a strong base, a concentrated external buffer, or a deliquescent salt/base additive could generate a local alkaline microenvironment or brine-like microphase.^101^ Because bulk pH cannot be applied directly to W0/W1 systems, B2 was assigned based on the presence of a strongly alkaline additive, a concentrated external base/buffer supply, or the potential for moisture uptake to form a locally basic liquid microphase. When the external base/buffer concentration was reported, conditions containing a strong or concentrated external base/buffer at ≥0.1 M were generally used as an operational guide for B2 assignment. Even in these cases, B2 does not indicate a uniformly high bulk-aqueous pH; it denotes a dry or interfacial operational regime with substantial external base availability.

Importantly, we applied the W/B-Class framework only to hydrolysis-dominant decontamination platforms in which water, surface hydroxyl groups, hydroxide ions, or catalyst-activated water primarily mediate bond cleavage at the P center. We excluded entries whose apparent performance was governed mainly by mechanisms other than water/base-dependent hydrolysis. For example, the silica sand/VX study examined environmental fate rather than a designed decontamination platform.^102^ The MgO@SBA-15@GO(IR)/DMNP system was better characterized as IR-assisted photothermal ethanolysis/transesterification in ethanol.^103^ The Ag–Z micromotor/DCP system relied directly on H_2_O_2_/SDS-driven propulsion and enhanced mixing,^60^ whereas the activated carbon/H_2_O_2_ system used H_2_O_2_ to induce perhydrolysis or oxidative post-treatment.^76^ We therefore excluded these cases from the core W/B-Class dataset.

Within this defined scope, W/B-Class functions as an operational screening framework that complements, rather than replaces, classifications based on catalyst type, humidity, pH, or reaction medium. It reinterprets literature data according to water availability, external base/buffer demand, wash-water use, and spent-liquid management rather than material class alone. This approach helps narrow the decontamination platforms that merit priority under realistic operational conditions.

#### 4.2.3. Distribution of reported performance across W/B-Class

Fig. 8 maps the literature-reported apparent decontamination performance of the hydrolysis-dominant core dataset after excluding the non-core cases defined in Section 4.2.2. Each data point was assigned according to the operational decision framework in Fig. 7. Table S1 lists the corresponding W/B-Class, the kinetic metric reported in the original source, the apparent *t*_90_, and the *t*_90_ bin used to determine marker size in Fig. 8. Marker shape denotes the decontaminant platform, marker fill distinguishes actual agents from simulants, and marker size denotes the binned apparent *t*_90_. The apparent *t*_90_ is a system-level descriptor used to place heterogeneous literature results on a common time scale; it does not represent intrinsic catalytic activity or an absolute reaction rate. Fig. 8 should therefore be interpreted as a map of data coverage and apparent-performance distributions across W/B regimes, not as an absolute ranking of materials. Small offsets among data points within the same W/B cell were introduced only to prevent marker overlap and carry no additional quantitative meaning. The figure is intended to show the overall distribution of reported performance and the extent of data coverage, whereas detailed comparisons under specific operational conditions are presented in Section 4.3.

The most prominent feature of Fig. 8 is the strong concentration of data in the W2 region. This bias largely reflects the relative ease of controlling experimental conditions and quantifying reaction performance in bulk-aqueous systems. In aqueous media, pH and buffer capacity can be defined more clearly, pseudo-first-order rate constants (*k*_obs_) can be derived more readily, and reaction products can be analyzed more systematically. Bulk water also provides a continuous supply of nucleophiles, including H_2_O and OH^−^, and can stabilize polar transition states through solvation. When a base or buffer is added, it can not only increase nucleophile availability but also neutralize acidic products and mitigate product-induced active-site deactivation.^100^ This bias also extends to agent identity. Many W2 entries involve G-series agents or related simulants because hydrolysis dominated by P–F bond cleavage can be followed relatively straightforwardly in aqueous media. By contrast, evaluation of V-series agents must resolve the competing P–S and P–O cleavage pathways and determine whether EA-2192 is formed, resulting in fewer directly comparable datasets.

This distribution also reflects the operating conditions favored by each decontaminant platform. Many MOF and POM entries occur in W2 because their reported reactivity often depends on hydrated reaction environments, solution-phase speciation, and external base or buffer conditions; for Zr-based platforms, Lewis-acidic metal centers and ligand-exchange processes are also central to catalysis. Zr-MOFs, MOF–fiber composites, and Zr-cluster/SBA-15 systems evaluated in 0.4–0.5 M NEM at pH ≥ 10 therefore cluster mainly in W2–B2 and exhibit short apparent *t*_90_ values.^44,77,83,86,104^ However, the W2 data are not distributed uniformly. Most of the shortest apparent *t*_90_ values occur in the base-rich W2–B2 regime, whereas W2–B0 and W2–B1 contain fewer entries. In the current dataset, W2–B1 is represented mainly by POM-catalyzed reactions in Tris–HCl. It should therefore be interpreted as an intermediate regime that illustrates the effects of solution speciation and buffer identity under moderate buffer conditions, rather than generalized as part of the broader high-performance MOF/NEM domain.^92^ Some MOF hydrogels and textile composites extend into W1–B0 by providing local water and built-in basic functionality without an external base input. By contrast, metal oxides and some zeolite/silica systems span a broader range of W0 and W1 conditions because fixed surface hydroxyl groups or defect sites can serve directly as reactive sites.

Conversely, the scarcity of W0 and W1 data reflects the difficulty of controlling experimental conditions and interpreting kinetics under water-limited conditions. In these regimes, reactions occur at heterogeneous solid interfaces rather than in a uniform aqueous phase. The observed apparent rate therefore depends not only on the intrinsic reactivity of the material but also on the amount and distribution of adsorbed water, mass transfer through thin water films, contaminant spreading, and product accumulation. Under W0 conditions, OH^−^ mobility and effective concentration cannot be defined in the same manner as in a bulk solution. In addition, deliquescent salts, concentrated bases, or salt-containing buffer additives can absorb moisture and generate local liquid microphases with brine-like characteristics. Data assigned to W0–B2 or W1–B2 therefore require particularly cautious interpretation.^101^

In summary, W2 conditions are useful for identifying the highest apparent performance achievable under controlled aqueous conditions. However, they do not directly represent the constraints of field decontamination, including limited water availability, restricted operational resources, the need to prevent further contaminant spread, and the management of secondary waste. Military decontamination doctrine distinguishes immediate, operational, and thorough decontamination according to purpose and available time and explicitly emphasizes contamination-spread control and waste management.^105,106^ Practical evaluation should therefore consider not only performance under optimized W2 conditions but also the extent to which apparent reactivity is retained as water and base availability shift toward more constrained regimes. On the basis of this interpretation and the distribution shown in Fig. 8, Section 4.3 discusses which decontamination platforms should be prioritized within each W/B regime under realistic operational constraints.

### 4.3. Selection criteria for hydrolysis-based decontaminants by W/B-Class

Based on the W/B-Class map in Fig. 8, this section discusses selection criteria in the main regimes where the available data support comparison across decontaminant classes. The literature data are not evenly distributed across all W/B regimes. Some regions contain only a few points, whereas others are dominated by specific material classes. We therefore focus on five regimes with sufficient reported data: W0–B0, W1–B0, W2–B0, W2–B1, and W2–B2. These regimes define two comparison axes. The first axis follows the effect of water availability without an external base or buffer: W0–B0, W1–B0, and W2–B0. The second axis follows the effect of external base/buffer availability under bulk-water conditions: W2–B0, W2–B1, and W2–B2. Rather than presenting a simple performance ranking, this analysis examines how each platform retains or loses apparent reactivity as water and base availability changes. It also considers what any loss in performance implies for field translation under increasingly constrained operating conditions. In the regime-specific discussion below, simulant-based results are treated as mechanistic screening evidence, whereas actual-agent results are treated as validation evidence for assessing field translation.^46,47^ For VX entries, the analysis considers not only apparent *t*_90_ but also EA-2192 detection status and, where reported, selectivity between P–S and P–O bond cleavage.^24^

**Fig. 9:**
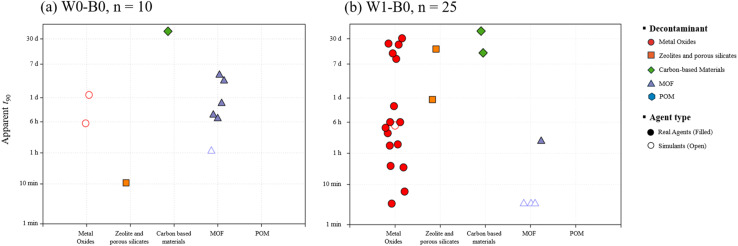
Apparent *t*_90_ distributions of hydrolysis-based decontaminants under (a) W0–B0 and (b) W1–B0 conditions. Marker shape indicates decontaminant class, and marker filling distinguishes actual agents from simulants. The *x*-axis positions were jittered within each material class only to avoid marker overlap and have no additional quantitative meaning.

Fig. 9 compares apparent *t*_90_ distributions under base-free, water-limited conditions. Fig. 9(a) represents W0–B0 conditions, where both external water and external base/buffer supply are absent or highly limited. Fig. 9(b) represents W1–B0 conditions, where adsorbed water, hydrated surfaces, or thin interfacial water layers are present, but no external base or buffer is supplied. These regimes are important because immediate or early-stage decontamination often occurs before bulk water, buffer, or liquid-recovery infrastructure is available.^105,106^ Thus, Fig. 9 provides a basis for evaluating decontaminants that can operate as dry barriers, reactive sorbents, or humid interfacial materials. However, these *t*_90_ distributions are intended only as a supplementary basis for interpreting reported apparent performance within the W0–B0 and W1–B0 regimes; they should not be used for direct intrinsic-activity comparisons with W2 or other bulk-aqueous datasets.

The W0–B0 region in Fig. 9(a) represents dry surface decontamination and reactive-barrier conditions. In this region, bulk water and an external OH^−^ reservoir are not available. Apparent performance therefore depends strongly on how effectively the material concentrates the agent at the surface or within pores and brings it into contact with limited surface hydroxyl groups or solid active sites. On an apparent *t*_90_ basis, the TiO_2_/MCM reactive adsorbent shows the shortest value in this regime, approximately 10.6 min for GB. Its loading-adjusted descriptor is also summarized as *k*_app_*V*_rxn_/*m*_decon_ = 1.73 × 10^−2^ L g^−1^ min^−1^. This behavior can be attributed to the ability of MCM-41 pores to concentrate the agent and to the dispersed TiO_2_ domains that act as reactive degradation sites for P–F or P–S bond cleavage.^70^ However, this result was obtained under hexane-based suspension conditions and should not be interpreted as direct evidence of absolute superiority under practical dry-surface conditions.

Among the W0–B0 entries for which loading-adjusted comparison is possible, dry Zr(OH)_4_ shows strong sensitivity to surface state and atmospheric exposure. For DMMP, *r*_app,m_ increased from 6.32 µmol g^−1^ min^−1^ under CO_2_ pre-dose conditions to 32.7 µmol g^−1^ min^−1^under N_2_-purged conditions. This trend suggests that surface carbonate formation or changes in surface hydroxyl availability can strongly affect dry reactions.^98^ By contrast, dry activated carbon is meaningful because it represents an actual VX entry, but its apparent *t*_90_ is approximately 66 970 min and its *r*_app,m_ is only 6.49 × 10^−3^ µmol g^−1^ min^−1^. It is therefore better interpreted as a slow sorbent or reference adsorbent than as a rapid dry decontaminant.^76^

MOF-based W0–B0 entries require two levels of interpretation. MOF-808/BPEIH/fiber shows relatively high reactivity toward DMNP, with an apparent *t*_90_ of 66.4 min and *r*_app,m_ = 394 µmol g_MOF_^−1^ min^−1^. However, this result is based on a simulant, and BPEIH provides intrinsic basic functionality within the composite. It should therefore not be generalized directly as actual dry-agent performance under W0–B0 conditions.^81^ In contrast, UiO-66-NH_2_ powder and MOFabric systems have higher field relevance because they were evaluated using GD droplets. MOFabric-19% and MOFabric-33% gave apparent *t*_90_ values of 535 and 435 min, respectively, which are shorter than that of UiO-66-NH_2_ powder. This improvement is consistent with the role of the PVDF nanofiber matrix in promoting GD droplet spreading and increasing the effective contact area with MOF particles.^84^ Because the active MOF mass was not clearly reported in the original source, the loading-adjusted descriptor was assigned as NC. The improvement should therefore be interpreted as enhanced contact efficiency from shaping rather than as an intrinsic increase in MOF catalytic activity.

Overall, TiO_2_/MCM appears fastest in W0–B0 from a kinetics-first perspective. For practical dry deployment, however, selection should also consider actual-agent validation, droplet spreading, active-site accessibility, shaping stability, and the availability of loading-adjusted comparisons.

The W1–B0 region in Fig. 9(b) represents humid or interfacial-water conditions without external base or buffer. In this regime, adsorbed water, hydration layers, pore-confined water, or thin interfacial water films are present, but a bulk aqueous phase is absent. Reactivity is therefore governed strongly by surface hydroxyl density, water distribution, agent spreading, and product accumulation. Metal oxide and hydroxide-like materials provide the largest number of actual-agent entries in W1–B0. Among them, Zr(OH)_4_ shows short apparent *t*_90_ values for GD and VX, 28.9 min and < 3.32 min, respectively. Its loading-adjusted descriptors are also summarized as *r*_app,m_ = 8.42 and > 224 µmol g^−1^ min^−1^, respectively, making Zr(OH)_4_ a compelling actual-agent candidate that retains high apparent reactivity under limited-water conditions.^50^ For VX, the desired detoxification pathway is P–S bond cleavage to EMPA and DESH, whereas P–O bond cleavage can generate the highly toxic byproduct EA-2192.^22,24^ The absence of detected EA-2192 over Zr(OH)_4_ therefore carries significance beyond reaction rate alone and identifies this system as an important example of VX P–S selectivity under W1–B0 conditions.

Hydrated TiO_2_-based materials are also promising W1–B0 candidates. nTiO_2_ gave an apparent *t*_90_ of 6.64 min for VX at 30 wt% H_2_O. Under no-wax 25 wt% H_2_O conditions, nTiO_2_ gave apparent *t*_90_ values of 26.6 min for VX and 259 min for GD. These results suggest that hydration layers and surface –OH/OH_2_ species on TiO_2_ can support nucleophile supply and P-center activation under interfacial-water conditions.^97^ However, the original studies did not clearly report *m*_decon_, so the loading-adjusted descriptors were assigned as NC. VX product selectivity was also not reported in sufficient detail. Thus, nTiO_2_ can be regarded as a fast W1–B0 candidate, but additional validation is needed before it can be considered a selective platform with confirmed EA-2192 suppression comparable to Zr(OH)_4_.

Other oxide entries show a clear agent-dependent performance gap. AP-Al_2_O_3_, nMgO, nCaO, and γ-Al_2_O_3_ degrade GD on minute-to-hour time scales, whereas their VX apparent *t*_90_ values generally extend to the day scale.^107–110^ For example, *n*MgO gives an apparent *t*_90_ of 93 min and *r*_app,m_ = 7.85 µmol g^−1^ min^−1^ for GD, but its VX performance decreases to an apparent *t*_90_ of 13 553 min and *r*_app,m_ = 3.70 × 10^−1^ µmol g^−1^ min^−1^.^106^ This contrast shows that G-series P–F bond cleavage under W1–B0 conditions cannot be generalized directly to V-series decontamination. Zeolite and carbon-based entries remain valuable because they include VX or GB actual-agent data, but their longer apparent *t*_90_ values and lower loading-adjusted descriptors make them more appropriate as reactive sorbents or reference adsorbents than as rapid interfacial decontaminants.^61,76^ Among MOF entries, NU-1000 gives an apparent *t*_90_ of 120 min and *r*_app,m_ = 17.4 µmol g^−1^ min^−1^ for GD at 50% RH, indicating that mesoporous Zr-MOFs can use limited interfacial water for actual-agent hydrolysis.^77^ By contrast, MOF-808/BPEIH powder and fiber systems show very short apparent *t*_90_ values for DMNP or DEMP simulants, but BPEIH supplies intrinsic basic functionality within the composite. These simulant-based results should therefore not be generalized directly to actual-agent performance.^81^ Overall, Zr(OH)_4_ and hydrated nTiO_2_ emerge as strong kinetics-first candidates in W1–B0, while Zr(OH)_4_ is the more convincing actual-agent selective platform because EA-2192 suppression was confirmed for VX.

Overall, Fig. 9 shows that W0–B0 and W1–B0 should not be treated as secondary regions simply because they contain fewer data than W2 conditions. These regimes capture conditions closer to dry or low-water decontamination, where bulk washing and external buffer supply are unavailable. When apparent *t*_90_ values and available loading-adjusted descriptors are considered together, TiO_2_/MCM shows a fast kinetics-first response in W0–B0, although its suspension-test condition limits direct translation to practical dry surfaces. In W1–B0, hydrated nTiO_2_ and, in particular, Zr(OH)_4_ emerge as more convincing actual-agent candidates; Zr(OH)_4_ is especially notable because EA-2192 suppression was confirmed for VX. From a deployment perspective, shaped systems such as MOFabric and internally functionalized MOF composites provide useful design directions for dry barriers and humid protective layers.

**Fig. 10:**
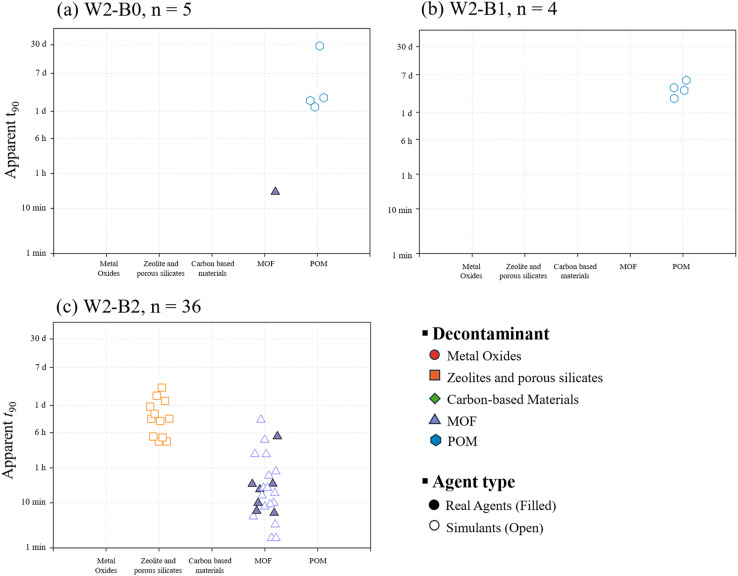
Apparent *t*_90_ distributions of hydrolysis-based decontaminants under bulk-aqueous W2 conditions: (a) W2–B0, (b) W2–B1, and (c) W2–B2. Marker shape indicates decontaminant class, and marker filling distinguishes actual agents from simulants. The *x*-axis positions were jittered within each material class only to avoid marker overlap and have no additional quantitative meaning. The plotted *t*_90_ values are apparent system-level time scales rather than intrinsic rate constants.

Fig. 10 compares apparent *t*_90_ distributions under bulk-aqueous W2 conditions by separating the data into W2–B0, W2–B1, and W2–B2 regimes. Whereas Fig. 9 shows performance distributions under dry and humid interfacial conditions with limited water supply, Fig. 10 examines how an external base/buffer reservoir changes apparent performance and product selectivity when continuous liquid water is present. Fig. 10 should therefore be read not as an intrinsic activity ranking under aqueous conditions but as a regime-level comparison for judging which platforms are better suited to water-only, moderate-buffer, or base-rich aqueous environments. In particular, W2–B0 and W2–B1 contain only a limited number of entries, whereas W2–B2 is dominated by data for Zr-MOFs, MOF–fiber composites, and Zr-cluster/SBA-15 systems.

Fig. 10(a) shows the W2–B0 region, where bulk water is present but no external base or buffer is added. The key question in this regime is whether a platform can retain hydrolytic reactivity and product selectivity in the presence of sufficient water but without an external basic reservoir. In the current dataset, UiO-67-N(Me)_2_ provides the most convincing actual-agent case. UiO-67-N(Me)_2_ shows an apparent *t*_90_ of 23.3 min for VX, and its loading-adjusted descriptor is summarized as.*k*_app_*V*_rxn_/*m*_decon_ = 2.97 × 10^−2^ L g^−1^ min^−1^.^83^ The absence of detected EA-2192 is especially important because it indicates more than rapid degradation; it shows that VX can be directed selectively toward P–S bond cleavage even without an external base or buffer. This selectivity can be attributed to the combined effects of Lewis-acidic activation at the Zr_6_ node and the local proton-transfer environment provided by the dimethylamino-functionalized linker.

POMs in W2–B0 are better interpreted as a comparative axis for understanding how buffer-free aqueous hydrolysis can occur at molecular active sites, rather than as actual-agent candidates that directly compete with UiO-67-N(Me)_2_. In D_2_O at pD 7 without buffer, the trisubstituted tungstoantimonate series shows decreasing apparent *t*_90_ values in the order {Gd_3_(HPO_3_)Sb_3_W_28_}, {Y_3_(HPO_3_)Sb_3_W_28_}, and {Yb_3_(HPO_3_)Sb_3_W_28_}, with values of 2889, 2495, and 1800 min, respectively. The lacunary {SbW_9_} precursor is much slower, with an apparent *t*_90_ of 40 898 min.^92^ This trend indicates that accessible Lewis-acid metal centers contribute to phosphoryl-group coordination and water activation. However, these results are based on the DMNP simulant, and the mass and volume conditions needed for loading-adjusted comparison were not sufficiently reported. Therefore, they are more useful for interpreting structure–reactivity relationships than for quantitative comparison with the VX result for UiO-67-N(Me)_2_.

Although W2–B0 contains relatively few entries, it provides an important benchmark for aqueous decontamination designs that minimize external base/buffer input. In this regime, UiO-67-N(Me)_2_ can be regarded as a strong base-free aqueous actual-agent candidate because it satisfies both rapid VX degradation and EA-2192 suppression. POMs, in contrast, show how Lewis-acidic molecular sites can provide reactivity under buffer-free conditions. The main selection criterion in W2–B0 is therefore not the single fastest apparent *t*_90_, but whether the catalyst structure itself can sustain water activation, proton transfer, P–S selectivity, and product release without an external base input.

Fig. 10(b) presents the apparent *t*_90_ distribution in the W2–B1 regime, where bulk water coexists with a moderate external buffer. In the current dataset, W2–B1 does not represent a broad MOF/NEM domain; it is composed mainly of POM-catalyzed DMNP hydrolysis data obtained in 125 mM Tris–HCl at pD 7. The apparent *t*_90_ values of these entries range from 2956 to 7574 min, and all fall within the 1–7 d bin.^92^ This regime should therefore be interpreted as a narrow intermediate regime that shows how buffer identity and solution speciation affect molecular POM catalysts under moderate buffer conditions, not as a generalized high-performance buffered regime for Zr-MOFs.

The POM data show that buffer species can strongly influence hydrolysis. Tris–HCl does more than regulate pH; it can participate in proton transfer or water activation. For example, the apparent *t*_90_ of the lacunary {SbW_9_} precursor decreased markedly from 40 898 min under no-buffer D_2_O conditions to 4515 min in Tris–HCl. In contrast, the relative activity trend among the metal-substituted tungstoantimonates was retained, with the Yb-substituted species remaining faster than the Y- and Gd-substituted analogues.^92^ These trends suggest that reactivity under moderate buffer conditions reflects not only the POM structure itself but also buffer participation and catalyst speciation in solution.

Thus, W2–B1 is meaningful as an intermediate regime between unbuffered W2–B0 and base-rich W2–B2. However, the current W2–B1 dataset lacks actual-agent entries, is concentrated in a specific POM system, and has loading-adjusted descriptors assigned as NC because the mass and volume information needed for normalization was not sufficiently reported. The main value of this regime is therefore not to identify the fastest candidate but to show how the active-site structure of molecular Lewis-acid catalysts, the proton-transfer environment, and buffer participation are coupled under near-neutral buffered conditions. Future studies should report rates, product distributions, and, for VX entries, EA-2192 suppression under the same moderate buffer/base conditions for actual G- and V-series agents. Such data would clarify the practical relevance of W2–B1 beyond the current POM/DMNP dataset.

Fig. 10(c) shows the W2–B2 regime, where bulk water coexists with a high-capacity external base/buffer reservoir. This region contains many entries for Zr-MOFs, MOF–fiber composites, and Zr-cluster/SBA-15 systems evaluated in 0.4–0.5 M NEM at pH ≥ 10, and the shortest apparent *t*_90_ values in Fig. 10 are concentrated mainly in this regime.^44,77,83,86,104^ This rapid response reflects the combined effects of Lewis-acidic activation at Zr_6_ nodes or Zr clusters, nucleophilic attack by node-bound water or hydroxide species, proton transfer in the NEM-rich medium, and buffering of acidic byproducts. The apparent *t*_90_ values in W2–B2 should therefore be interpreted as system-level responses that couple catalyst structure with base-rich assay conditions.

Among MOF-based entries, UiO-67 derivatives, NU-1000, MOF-808/PET, and UiO-66-NH_2_/PET show fast apparent responses. UiO-67-N(Me)_2_ gives an apparent *t*_90_ of 5.98 min for VX, with no detected EA-2192, making it an actual-agent example that retains P–S selectivity even under W2–B2 conditions.^83^ NU-1000 gives an apparent *t*_90_ of 9.97 min for GD, whereas MOF-808/PET and UiO-66-NH_2_/PET show minute-scale reactivity toward both DMNP and GD, demonstrating that powder-level Zr-MOF activity can be translated into fibrous forms.^77,86^ At the same time, UiO-66-NH_2_ powder shows shorter apparent *t*_90_ values as catalyst amount increases, decreasing from 121 min to 21.6 min and then to 8.3 min.^85^ Thus, the fast values in W2–B2 reflect not only active-site structure but also catalyst loading and assay design.

Zr-cluster/SBA-15 systems generally show longer apparent *t*_90_ values than Zr-MOFs, but they provide a useful platform for separating the effects of Zr_6_ or Zr_12_ cluster structure, ligand identity, support acidity, and pore accessibility on a mesoporous silica support.^104^ Zr_12_-acetic acid-P-SBA-15 and SBA-15 + Zr_12_-acetic acid show relatively short *t*_90_ values, whereas sulfonic-acid-functionalized SBA-15 and some Zr-SBA-15 systems require longer reaction times. These differences show that W2–B2 reactivity is not determined by the presence of Zr centers alone. Cluster nuclearity, ligand environment, support acidity, and pore accessibility also contribute to the observed apparent performance.

Accordingly, W2–B2 candidates should not be judged by the fastest apparent *t*_90_ alone. Selection should also consider the hydrolysis response under base-rich aqueous conditions, loading-adjusted descriptors where calculable, EA-2192 suppression for VX entries, and whether powder-level activity is retained in fiber, textile, or supported forms. By these criteria, Zr-MOFs are the central practical candidates in W2–B2, whereas Zr-cluster/SBA-15 systems serve as comparative platforms for supported Zr active-site design and pore-accessibility effects. However, W2–B2 performance requires NEM or other base/buffer input, spent-liquid recovery, neutralization, and wastewater treatment. Fast aqueous reactivity should therefore be interpreted together with the operational cost of implementing a base-rich treatment regime.

Overall, the W/B-Class analysis is not a ranking system based on a single fastest apparent *t*_90_ value. It is an interpretive framework for identifying the platforms that are most defensible under different water/base operating regimes. In W0–B0, TiO_2_/MCM shows a fast kinetics-first response, but its suspension-test condition must be considered; from the perspective of dry deployment, shaped barrier systems such as high-loading MOFabric are also important candidates.^70,84^ In W1–B0, hydrated nTiO_2_ and Zr(OH)_4_ emerge as promising low-water decontamination candidates, and Zr(OH)_4_ is especially compelling because EA-2192 suppression was confirmed for VX.^50,97^ In W2–B0, UiO-67-N(Me)_2_ represents the key base-free aqueous actual-agent case because it combines fast selective VX hydrolysis with EA-2192 suppression without an external base or buffer.^83^ W2–B1 is currently limited mainly to POM/Tris–HCl systems, but it serves as an intermediate regime that isolates the effects of moderate buffer chemistry and solution speciation on molecular catalyst reactivity.^92^ In W2–B2, Zr-based MOFs such as UiO-67 derivatives, NU-1000, MOF-808/PET, and UiO-66-NH_2_/PET occupy the fastest apparent *t*_90_ region, but this performance also reflects base-rich aqueous assay conditions such as 0.4–0.5 M NEM.^77,83,86^ Therefore, the suitability of a decontamination platform should be judged not by the fastest apparent *t*_90_ alone but by how well it balances actual-agent validation, loading-adjusted response, suppression of toxic byproducts such as EA-2192, shaped-form applicability, and post-treatment burden within the intended W/B regime.

## 5. Life-cycle considerations for hydrolysis-based decontaminants

### 5.1. Screening-level cradle-to-gate LCA of decontaminants

The sustainability of decontaminant platforms cannot be judged by reaction rate or removal efficiency alone. Their environmental burden begins during production stages such as raw material extraction, precursor production, synthesis, washing, and drying. During practical decontamination, additional operational burdens can arise from water supply, external base/buffer input, spent-sorbent handling, decontamination-liquor recovery, neutralization, and wastewater treatment.^111,112^ A life-cycle perspective is therefore needed to connect production-stage environmental burdens with operational resource requirements and waste-management burdens.^113,114^

Before the 2000s, early studies mainly focused on life cycle inventory (LCI), which lists and quantifies material and energy flows during manufacturing.^115^ Recent LCA studies have moved beyond inventory analysis and now consider multiple impact categories, including climate change, human toxicity, ecotoxicity, and water scarcity. These studies show that environmental impacts can vary substantially even within the same material class. Key process variables include raw material and precursor selection, synthesis route, production scale, solvent use, washing conditions, and energy source.^116,117^

The LCA discussion in this section does not recalculate results from different studies into a single complete comparative LCA. Instead, it provides a screening-level life-cycle interpretation that links the production-stage burdens of decontaminant materials with the resource inputs actually used in decontamination experiments. We follow the basic structure of ISO 14040 and ISO 14044, including goal and scope definition, life-cycle inventory, life-cycle impact assessment, and interpretation. However, because the cited studies differ in functional unit, system boundary, production scale, and LCIA method, we do not perform an absolute material ranking.^118,119^

Table 5 summarizes cradle-to-gate LCA data for decontaminant-relevant material classes. The table covers production stages from raw material extraction to the factory gate and lists the functional unit or comparison basis used in each cited study. Climate-change indicators, such as global warming potential (GWP) or CO_2_-eq, were used as the main common basis when available. Other reported indicators were retained when they helped identify dominant process burdens. POM-based decontaminants were excluded from the quantitative comparison in Table 5 because directly comparable cradle-to-gate LCA studies remain insufficient. Therefore, the kg CO_2_-eq kg-material^−1^ values in Table 5 should not be interpreted as a ranking of decontamination performance. They should instead be considered together with the amount of material used under actual decontamination conditions, water consumption, external base/buffer input, and spent-liquid generation. This connection between W/B-Class interpretation and LCA is discussed in the following section.

**Table 5:** Screening-level cradle-to-gate LCA data for decontaminant-relevant material classes. Reported values were compiled from studies with different functional units, system boundaries, production scales, and impact-assessment methods

Decontaminant	Functional unit	Comparison basis	Cradle-to-gate metrics	Dominant burden drivers	Ref.
Metal oxide (CaO; hydrated lime)	1 kg hydrated lime; cradle-to-gate	Electricity source and kiln fuel type	GWP 0.94 kg CO_2_-eq per kg; fuel switch up to −22%	Limestone decomposition, kiln fuel use, and electricity consumption	120
Metal oxides (MgO)	1 t magnesia refractory raw material; production-stage GHG basis	Product grade and thermal processing intensity	2.7–7.8 t CO_2_-eq/t	Process emissions, calcination, fusion, and product grade	121
Zeolites (Zeolite A)	1 kton Zeolite A product; scaled batch and continuous-flow systems	Production mode and recirculation strategy	Lower impacts in continuous-flow; NaOH major contributor	NaOH consumption, recirculation system, production mode, and process scale	122
Zeolites (NaA, NaY, FEZSM-5)	1 t zeolite product; reported as kg CO_2_-eq kg^−1^ and MJ kg^−1^	Precursor source and zeolite type	NaA 3.64/1.61, NaY 3.61/1.31, FeZSM-5 3.25/0.882 kg CO_2_-eq per kg; PED 54.5/25.4, 54.8/20.3, 64.4/15.6 MJ kg^−1^	Chemical precursors or natural minerals, NaOH, washing water, and electricity	123
Porous silicates (mesoporous silica)	1 kg mesoporous silica material; small- and large-scale synthesis	Production scale	54 ± 30, 31 ± 18 kg CO_2_-eq per kg	Solvents, TEOS, structure-directing agents, calcination, and extraction	124
Activated carbon (coal, coconut shell, wood, peat, and reactivated coal AC)	1 kg GAC or 1 t AC, depending on source; cradle-to-gate	Feedstock type, production route, and reactivation	Coal AC highest in 10/12 categories; reactivated coal AC 72–80% lower GWP; woody-biomass AC CED 119.5 MJ kg^−1^, GWP 8.96 kg CO_2_-eq per kg	Feedstock, direct emissions, electricity, carbonization, activation, and reactivation route	125 and 126
MOFs (ZIF-8)	1 kg produced ZIF-8 powder; cradle-to-gate synthesis routes	Solvent system, washing demand, and electricity mix	86.60–1571.16 kg CO_2_-eq per kg; electricity 60.25–580.7 kWh kg; heat 68–1605 MJ kg; DMF/MeOH > 85%; electricity up to 13%	Solvent demand, repeated washing, drying, and electricity consumption	127
MOFs (UiO-66-NH_2_)	1 kg UiO-66-NH_2_ product; pilot-scale cradle-to-gate production	Synthesis route	43 kg CO_2_-eq per kg; aqueous route up to −91%	Synthesis route, organic-solvent use, precursor demand, washing, and drying	128

For metal oxides, the dominant production-stage burdens arise from high-temperature calcination, fusion, thermal energy input, and electricity consumption. CaO-derived hydrated lime has been reported to have a cradle-to-gate GWP of approximately 0.94 kg CO_2_-eq kg^−1 121^. MgO products show a broader range, from 2.7 t CO_2_-eq t^−1^ for caustic calcined magnesia to 7.8 t CO_2_-eq t^−1^ for two-phase fused magnesia.^121^ This difference reflects product grade and process intensity. Thus, the production-stage burden of metal oxides depends less on the oxide class itself than on calcination conditions, fusion steps, and energy mix.

For zeolites and porous silicas, precursor sourcing and process design are the main burden drivers. In Zeolite A production, continuous-flow production was generally more favorable than batch production, although the benefit differed across impact categories. The recirculation system was identified as a key target for further improvement, while upstream burdens from NaOH and aluminum hydroxide remained substantial.^122^ Precursor sourcing also affects the environmental profile. Zeolite synthesis from natural minerals can reduce resource-related burdens compared with synthesis from chemical precursors.^123^ Mesoporous silica shows a similar process-dependent profile. Its impacts depend strongly on the synthesis procedure, especially template removal, solvent use, and calcination or extraction steps.^124^ Renewable energy use and solvent-recovery steps, such as distillation or rectification, have therefore been proposed as mitigation strategies.

Activated carbon shows large variations in environmental burden depending on feedstock and regeneration route. LCA studies comparing coal-derived, coconut-shell-derived, wood-derived, peat-derived, and reactivated activated carbons show that direct emissions, electricity consumption, carbonization, activation, and reactivation strongly affect the final burden.^125,126^ Coal-based virgin activated carbon showed the highest burden in many impact categories, whereas reactivated coal-based activated carbon showed a substantially lower GWP than virgin activated carbon when biogenic emissions were included.^126^ Wood-based activated carbon also showed that natural gas, LPG, and electricity inputs during carbonization and activation are major burden drivers.^125^ Therefore, the production-stage environmental profile of activated carbon is governed mainly by feedstock selection, activation route, regeneration strategy, and energy mix.

For MOFs, solvent use, precursor demand, and washing steps dominate production-stage burdens. In ZIF-8 production, routes using DMF and MeOH accounted for more than 85% of the total environmental impact, while electricity contributed up to 13% in some routes.^127^ For UiO-66-NH_2_, the aqueous route was reported to have a carbon footprint of 43 kg CO_2_-eq kg^−1^. Replacing the solvothermal route with an aqueous route could reduce the overall environmental burden by up to 91%.^128^ These results show that the production-stage burden of MOFs is governed more by synthesis route, solvent choice, washing-step design, and solvent recovery than by MOF topology alone.^129^

In summary, the production-stage environmental profile of a decontaminant is governed by the process variables that dominate the burden within each material class. Materials such as CaO, mineral-derived zeolites, and reactivated activated carbon can have relatively simple supply chains and lower production-stage burdens. These materials are therefore better suited for large-scale, expendable, or preliminary containment uses, including surface decontamination powders, slurry-type treatment, and sorbent fills.^120,126^ By contrast, MgO, mesoporous silica, and MOF-based decontaminants generally carry higher production burdens, but they offer advantages in structural control, active-site design, and functional integration.^124,128^ These materials may be more appropriate for high-value applications where small material loadings can deliver selective or concentrated performance, such as respirator canisters, collective-protection filters, reactive coatings, and protective textiles. Applicability should therefore be judged by matching each decontaminant's production burden and functional strength with the intended treatment scale, application form, contaminated medium, water/base regime, and waste-management capacity.

### 5.2. W/B-class interpretation of operational burdens

The cradle-to-gate LCA summarized in Section 5.1 is useful for comparing production-stage burdens of decontaminant-relevant material classes. However, it has limited ability to describe the practical applicability of each platform by itself. Even for the same material, the required amount of wash water, buffer or strong base, recovery system, and post-treatment capacity can vary with the W/B-Class regime. This section therefore links the production-stage burdens in Table 5 with the W/B-Class interpretation in Section 4 and the loading data in Table S2 to examine resource inputs and waste-management burdens in each regime. From this perspective, dry and low-water regimes reduce water and liquid-waste burdens but make production-stage burden and spent-solid handling more important. In contrast, bulk-aqueous regimes place greater emphasis on reaction-liquid volume, buffer/base input, and wastewater treatment. This interpretation can also be connected to operational stages that distinguish immediate, operational, thorough, and clearance decontamination.^105,106^

To compare operational burdens across W/B-Class regimes more quantitatively, we used a 90% destruction basis as a common calculation basis. This basis compares the amount of decontaminant, reaction liquid, or external base/buffer required to destroy 90% of 1 mmol of the agent or simulant under the reported conditions. The amount destroyed was therefore defined as *n*_destroyed_ = 0.90*n*_agent,0_. Each resource intensity was calculated according to eqn (5).5
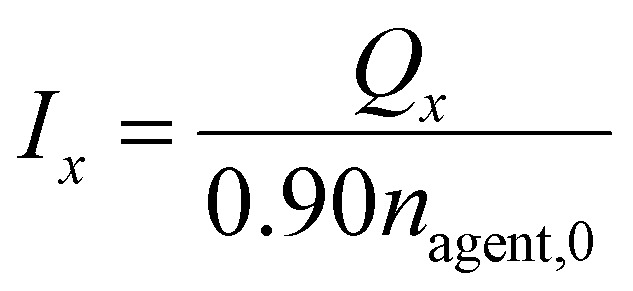
where *Q*_*x*_ denotes the decontaminant mass (*m*_decon_), reaction-liquid volume (*V*_rxn_), or external base/buffer amount (*C*_base_*V*_rxn_). Accordingly, *I*_decon_, *I*_liq_, and *I*_base_ are expressed as g-decontaminant mmol-agent-destroyed^−1^, mL mmol-agent-destroyed^−1^, and mol-base mol-agent-destroyed^−1^, respectively. This calculation is not a full comparative LCA. Instead, it serves as a screening-level resource inventory that links the experimental conditions in Table S2 with the cradle-to-gate burdens summarized in Table 5.

From this perspective, W0–B0 and W1–B0 can be interpreted as regimes closer to immediate or operational decontamination before bulk washing or external base/buffer supply becomes available. As discussed in Section 4.3, the key criterion in these regimes is not the maximum reaction rate under optimized aqueous conditions but whether the material can initiate surface-mediated reactions under limited water availability. Accordingly, structured dry barriers and reactive sorbents are relatively defensible candidates in W0–B0, whereas hydrated oxides and sorbent–catalyst composites become important in W1–B0. However, the burden in these regimes shifts from liquid waste toward solid decontaminant loading and spent-solid handling. For example, the Zr(OH)_4_/VX W1–B0 entry in Table S2 corresponds to 0.234 g of Zr(OH)_4_ and 75.6 µmol of VX. On a 90% destruction basis, this gives *I*_decon_ ≈ 3.44 gZr(OH)_4_/mmol VX destroyed. This value indicates a much larger solid input than typical W2 aqueous MOF entries, but it also illustrates the W0/W1 trade-off: external base/buffer input and bulk spent-liquid generation can be reduced at the expense of higher solid use and spent-solid management.

Operational and environmental applicability cannot be explained by emissions per kilogram of material alone. It must also account for water demand, auxiliary chemical input, spent-solid handling, spent-liquid recovery, neutralization, and downstream treatment. Military decontamination doctrine provides a useful operational analogy because it classifies decontamination by urgency, available time, and resource constraints.^105,106^ The same W/B-Class can also be extended to emergency response, contaminated-infrastructure clearance, hazardous-waste handling, and environmental remediation.

W0–B0 and W1–B0 are low-water regimes. They are relevant to immediate response, dry surface treatment, protective barriers, and preliminary containment of contaminated solids or porous media. According to FM 3–11.5, immediate decontamination prioritizes life protection and contamination-spread control; skin decontamination should be performed within 1 min, and personal equipment decontamination within 15 min.^105,106^ In these regimes, the key requirement is not the maximum apparent rate under ideal aqueous conditions. It is the ability to initiate degradation at dry or humid interfaces while limiting water use, liquid waste, and logistical demand.

From a life-cycle perspective, W0–B0 and W1–B0 reduce wash-water demand, base/buffer input, and spent-liquid recovery. However, they shift the burden toward material production, spent-solid handling, and verification of residual toxicity. Materials such as CaO and reactivated activated carbon can be favorable for expendable or preliminary containment uses because they have relatively simple supply chains and low production-stage burdens.^120,126^ Mineral-derived zeolites can also reduce precursor-related burdens compared with zeolites synthesized from chemical precursors.^123^ These advantages do not eliminate waste-management concerns. Used powders, reactive sorbents, filters, and protective textiles may still contain adsorbed agents, partially degraded organophosphorus species, or toxic byproducts. Thus, the practical value of W0/W1 systems depends on retained reactivity, product selectivity, and safe spent-solid management.

W2–B0 can be interpreted as a regime closer to thorough decontamination because bulk water is available. However, because this regime corresponds to water-only aqueous conditions without an added external buffer or base, it requires wash-water supply and recovery but imposes a relatively small burden from additional chemical input.^106^ As discussed in Section 4.3, the key question in W2–B0 is not simply whether hydrolysis can occur in water. Rather, it is whether selective hydrolysis and catalytic turnover can be maintained for actual agents under unbuffered conditions. In this respect, Zr-based MOFs such as UiO-67-N(Me)_2_ provide an important example because they suppress EA-2192 formation and promote selective VX hydrolysis without an external buffer or base.^83^ Therefore, the applicability of W2–B0 should be judged by whether selective aqueous decontamination of actual agents can be achieved without additional base assistance, even when the production-stage burden of the material is relatively high. The UiO-67-N(Me)_2_/VX entry in W2–B0 corresponds to *I*_decon_ ≈ 0.189 g per mmol VX destroyed and *I*_liq_ ≈ 56.7 mL per mmol VX destroyed. These values provide a water-only benchmark for comparison with the W2–B2 condition discussed below.

By comparison, W2–B1 represents bulk-aqueous conditions with a weak or moderate buffer. This regime requires limited buffer/base supply and wastewater management in addition to wash-water recovery, and therefore carries a higher operational burden than W2–B0. In the current dataset, however, W2–B1 does not represent high-performance Zr-MOF/NEM conditions; it is represented mainly by POM/Tris–HCl systems.^92^ W2–B1 is therefore best interpreted as an intermediate regime for evaluating how buffer identity and solution speciation affect molecular catalyst reactivity under controlled aqueous conditions with limited buffer/base support. The representative W2–B1 condition in Table S2 uses 4.2 mM DMNP and 125 mM Tris–HCl. On a concentration basis, applying the 90% destruction criterion gives a Tris reservoir of approximately 33.1 mol Tris/mol DMNP-destroyed.

W2–B2 contains many of the fastest apparent performance values, but this does not mean that it is automatically the most suitable regime for practical deployment. Performance in this regime reflects not only intrinsic catalyst activity but also the acceleration provided by a strong base or high-concentration buffer reservoir. A contrasting design strategy is illustrated by Im@MOF-808, in which imidazole base functionality is incorporated inside the pores of MOF-808 to promote DMNP hydrolysis in pure water and high-humidity conditions without an external liquid base.^94^ This approach can be interpreted as a way to reduce dependence on the external base/buffer reservoir that characterizes W2–B2. Therefore, W2–B2 performance should be evaluated together with downstream burdens such as chemical supply, wash-liquid recovery, neutralization, and treatment of corrosive or salt-rich effluents. CBRN decontamination wastewater has been reported as a complex effluent containing decontaminants, wash water, contaminants, solids, and organic matter.^111^ Strongly alkaline decontamination fluids can also be difficult to treat because of their corrosivity and complex composition.^112^ W2–B2 is therefore more appropriate for clearance decontamination, equipment washing, adsorbent regeneration, or downstream treatment processes where sufficient material supply and post-treatment infrastructure are available, rather than for immediate forward decontamination.^106^

This trade-off can be quantified using the UiO-67-N(Me)_2_/VX system, for which the same catalyst and agent were reported under both W2–B0 and W2–B2 conditions. According to Table S2, both entries used 2.5 mg of UiO-67-N(Me)_2_, 14.7 µmol of VX, and a total reaction volume of approximately 0.75 mL. On a 90% destruction basis, the amount of destroyed VX is 13.23 µmol, giving the same *I*_decon_ of 0.189 g per mmol VX-destroyed and the same *I*_liq_ of 56.7 mL per mmol VX-destroyed in both regimes. However, the W2–B2 condition used 0.5 M NEM at pH 10, corresponding to an additional 0.375 mmol of NEM. This is equivalent to 28.3 mol-NEM/mol VX-destroyed, or 3.26 g-NEM/mmol VX-destroyed when the molecular weight of NEM is taken as 115.18 g mol^−1^. As a result, the apparent *t*_90_ decreases by approximately 3.9-fold, from 23.3 min in W2–B0 to 5.98 min in W2–B2, but this improvement requires a large external base/buffer reservoir and introduces additional post-treatment burden.

These results do not mean that W2–B2 is intrinsically superior to W2–B0. Rather, they show that rapid aqueous decontamination can be achieved when sufficient external base/buffer is available. At the same UiO-67-N(Me)_2_ loading and the same reaction-liquid volume, W2–B2 shortened the apparent *t*_90_ from 23.3 to 5.98 min, but this improvement occurred with the introduction of a 0.5 M NEM reservoir. W2–B2 should therefore be interpreted as a regime suited to controlled aqueous treatment when rapid decontamination is prioritized and systems for wash-liquid recovery, neutralization, and wastewater treatment are available. By contrast, W2–B0 is slower but provides a low-chemical-input aqueous benchmark because it achieved VX degradation with no detected EA-2192 under the reported analytical conditions without an external base or buffer. The key point of W/B-Class-guided selection is therefore not to choose the shortest *t*_90_, but to balance the target decontamination time, water/base availability, and secondary-waste management burden.

Linking the cradle-to-gate GWP values in Table 5 with the loading data in Table S2 provides an order-of-magnitude estimate of the material-production burden in this example. Because no direct LCA value is available for UiO-67-N(Me)_2_, the value reported for aqueous-route UiO-66-NH_2_, 43 kg CO_2_-eq kg-material^−1^, can be used as a Zr-MOF proxy. This gives an estimated catalyst-production burden of approximately 8.1 g CO_2_-eq mmol^−1^ VX-destroyed. However, this value applies equally to both W2–B0 and W2–B2 because the same catalyst loading was used. It is therefore not the main factor that differentiates the two regimes. The practical difference arises from the NEM reservoir added in W2–B2 and the resulting spent-liquid treatment burden. Because NEM-specific emission factors and wastewater-treatment inventories are not yet available for this comparison, the additional W2–B2 burden should be reported as external base/buffer intensity rather than converted into a definitive LCIA ranking.

**Fig. 11:**
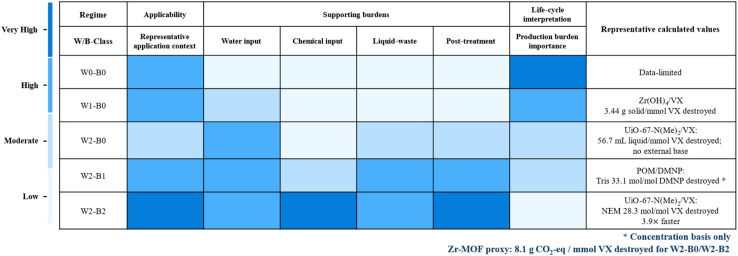
Resource-burden map across W/B-Class regimes. Color intensity indicates the relative degree of operational fit, supporting burdens, and production-burden importance. Representative calculated values were derived from Table S2 on a 90% destruction basis where sufficient loading information was available. The Zr-MOF proxy value links the UiO-67-N(Me)_2_/VX catalyst loading with the cradle-to-gate GWP metric in Table 5. The figure summarizes how the dominant burden shifts among solid decontaminant use, water/liquid demand, external base/buffer input, liquid-waste generation, and post-treatment requirements.

Fig. 11 presents a resource-burden map showing how the main resource inputs and post-treatment burdens shift across W/B-Class regimes. The color scale indicates the relative importance of each burden category, and the final column lists representative resource-intensity values calculated on a 90% destruction basis from the entries in Table S2. This map shows that solid decontaminant loading and spent-solid handling become major considerations in W1–B0, whereas external base/buffer input and post-treatment burden dominate in W2–B2. This contrast demonstrates that W/B-Class can serve not only as a performance-classification scheme but also as a practical guide for distinguishing application scenarios. For example, W1–B0 is relevant to dry or low-water surface decontamination where water supply and liquid recovery are limited, but this regime requires careful consideration of solid-decontaminant loading, recovery, and disposal after use. By contrast, W2–B0 provides a benchmark for aqueous treatment with reduced external base use, whereas W2–B2 is better interpreted as a regime suited to rapid treatment when wash-liquid recovery, neutralization, and wastewater-treatment infrastructure are available.

Future decontaminant design should therefore move beyond the pursuit of the shortest apparent *t*_90_. It should also aim to reduce the water input, external base/buffer input, solid loading, and liquid-waste treatment burden required to achieve a given level of decontamination performance. To retain the rapid reactivity associated with W2–B2 while reducing dependence on external base/buffer reservoirs, promising strategies include internal-base functionality, local water management, shaped sorbent–catalyst architectures, and reusable decontamination platforms. If future studies report *m*_decon_, *n*_agent_, *V*_rxn_, base/buffer concentration, number of reuse cycles, and spent-liquid treatment volume together with kinetic and product-selectivity data, W/B-Class can move beyond literature classification and serve as a pre-screening criterion that jointly considers decontamination performance, resource input, and secondary-waste burden.

## 6. Conclusions and future perspectives

NA decontamination cannot be reduced to selecting the material with the fastest reaction rate. It is a system-level problem that requires simultaneous consideration of agent identity, water/base availability, reaction selectivity, contaminated-media characteristics, and the burdens associated with production, operation, and waste treatment. This review compared hydrolysis-based decontamination platforms based on metal oxides, zeolites and porous silicas, carbon-based composites, MOFs, and POMs. By reinterpreting these platforms according to W-Class and B-Class, it sought to connect literature-reported reaction data with practical operational environments.

Under dry or low-moisture conditions, oxide-based catalysts and structured dry barriers showed favorable characteristics for immediate and operational decontamination. Under bulk-water conditions, Zr-based MOFs showed strengths in selective aqueous decontamination and suppression of toxic byproducts. Strong-base conditions can provide very rapid kinetic metrics, but they also require high chemical inputs and substantial post-treatment capacity. These conditions are therefore better interpreted as controlled treatment regimes. Ultimately, the suitability of each platform depends on water and base availability and mission level. Decontaminant selection should match the required hydrolytic performance with the water/base resources, contaminated medium, application form, and waste-management capacity of each operational context.

The practical effectiveness of decontaminant materials can be assessed more reliably when production-stage burdens and W/B-Class-specific operational burdens are considered together. In this review, the LCA-based quantitative comparison was limited to cradle-to-gate production stages for which cross-literature comparison was possible. Water demand, auxiliary chemical input, spent-solid handling, spent-liquid recovery, and post-treatment burden during use were instead interpreted as W/B-Class-linked resource and waste-management requirements. Because the decontamination benefit and the associated burden can change with the W/B condition even for the same material, evaluation of catalytic hydrolysis technologies should move beyond laboratory reactivity comparisons toward system-level assessment that reflects real military operating conditions and resource constraints.

Several data gaps must be addressed before this W/B-Class-based interpretation can be applied more precisely. For A-series agents, publicly available hydrolysis kinetics, product distributions, and comparable test-condition data remain insufficient. If comparable datasets become available for A-series agents across W/B-Class conditions, the present framework could be extended to evaluate their surface persistence, low-water reactivity, and byproduct behavior on the same basis used for G- and V-series agents. POMs are also mechanistically important platforms, but their practical relevance requires more systematic validation because actual-agent performance data, comparable cradle-to-gate production data, and W/B-Class-specific operational-burden data remain limited. In addition, the W0–B1, W0–B2, and W1–B1 regions, which remain sparsely populated in the current W/B mapping, should be supplemented with data obtained using actual agents or well-validated simulants. Future studies should also report product selectivity, toxic-byproduct suppression, long-term stability of shaped bodies and composites, use-phase resource inputs, and downstream treatment burdens for each W/B-Class regime. As these datasets expand, W/B-Class analysis will become more precise and will better support realistic decontaminant design and military application strategies.

Once such data are accumulated, the W/B-Class framework can move beyond literature classification and connect with computational chemistry and data-driven material design. DFT calculations and structure–activity relationship analyses can provide molecular descriptors such as Lewis acidity of metal nodes, defect density, node-bound water population, and substrate/product binding energies. If these descriptors are organized together with apparent *t*_90_, loading-adjusted descriptors, EA-2192 outcomes, and water/base inputs, they can support AI/ML-guided catalyst screening. Emerging platforms, including defect-engineered materials, single-atom catalysts, COFs, MXenes, nanozymes, and hybrid multifunctional architectures, can further integrate adsorption, local water/base management, hydrolysis, and regeneration within a single material system. Recent studies on emerging porous, two-dimensional, and enzyme-mimetic catalytic materials support this broader direction, although most platforms still require actual-agent validation before field translation.^130^ However, for these platforms to advance into practical decontamination technologies, they must be evaluated not only by actual-agent validation but also by repeated-use stability, shaped-body durability, active-site regeneration, spent-material handling, cradle-to-gate production burden, use-phase resource input, and post-treatment burden.

Ultimately, hydrolysis-based decontaminant design should move beyond achieving the fastest laboratory *t*_90_. It should optimize performance, selectivity, durability, regenerability, manufacturing burden, and waste-treatment feasibility under realistic W/B-Class conditions. From a scale-up perspective, continuous or scalable production, shaping yield, binder consumption, batch-to-batch reproducibility, and material throughput should also be considered. These metrics will determine whether the high reactivity of powder catalysts can be translated into practical filters, textiles, coatings, or reusable modules. The W/B-Class framework proposed in this review can therefore serve not simply as a performance-comparison tool but as a system-level decision framework for designing operationally viable decontamination platforms by integrating water/base availability, detoxification selectivity, application form, resource input, and post-treatment burden.

## Conflicts of interest

There are no conflicts to declare.

## Supplementary Material

RA-OLF-D6RA04543J-s001

## Data Availability

No primary research results, software or code have been included and no new data were generated or analyzed as part of this review. The literature-derived quantitative data supporting Fig. 8–10, including W/B-Class assignments, original kinetic metrics, apparent *t*_90_ values, loading-adjusted descriptors, and EA-2192 assessment status, are provided in Tables S1 and S2 of the supplementary information (SI). Supplementary information: Table S1. Tabulated W/B-Class assignment and apparent *t*_90_ dataset used for Fig. 8–10. Table S2. Experimental conditions and loading-adjusted apparent kinetic descriptors for the entries in Table S1, including EA-2192 outcomes for VX-containing entries. NC, not calculated; NR, not reported in the original source; N/A, non-VX entry. For EA-2192 outcomes, “Not detected” means that EA-2192 was not detected under the reported analytical conditions, whereas “Not assessed” means that EA-2192 formation was not evaluated or not reported. See DOI: https://doi.org/10.1039/d6ra04543j.
