# Supplementary material for: Hydrolysis-based degradation of organophosphorus nerve agents and simulants: water/base-dependent reactivity, catalytic materials and life-cycle considerations
Source: RSC Adv. 2026 Aug 6;16(39):43128–60. doi: 10.1039/d6ra04543j (PMC13444696; doi:10.1039/d6ra04543j)
Supplement: Supplementary file 1 [file RA-016-D6RA04543J-s001.pdf]

Supplementary Information (SI) for RSC Advances.
This journal is © The Royal Society of Chemistry 2026

Supplementary information

Table S1. Tabulated W/B-Class assignment and apparent t_{90} dataset used for Figures 8–10.

No.	Ref.	Decontaminant	Specific Decontaminant	Agents	W/B-Class	Reported values	t_{90} (min)	t_{90} bin
1	98	Metal hydroxide / oxide-like	$Zr(OH)_4$ (dry CO_2 pre-dose)	DMMP	W0-B0	k_{obs} = 0.00137 min^{-1}	1,681	1–7 d
2	98	Metal hydroxide / oxide-like	$Zr(OH)_4$ (dry N_2-purged)	DMMP	W0-B0	k_{obs} = 0.00708 min^{-1}	325	1–6 h
3	70	Zeolites & porous silicates	TiO_2/MCM reactive adsorbent	GB	W0-B0	$t_{1/2}$ = 3.2 min	10.6	≤ 1 h
4	76	Carbon-based materials & composites	Activated carbon (S-AC), dry	VX	W0-B0	$t_{1/2}$ = 14 d	66,970	> 7 d
5	84	MOF	UiO-66-NH_2 powder	GD	W0-B0	$t_{1/2}$ = 315 min	1,046	6–24 h
6	84	MOF	MOFabric-7% (PVDF/UiO-66-NH_2)	GD	W0-B0	$t_{1/2}$ = 1,616 min	5,368	1–7 d
7	84	MOF	MOFabric-13% (PVDF/UiO-66-NH_2)	GD	W0-B0	$t_{1/2}$ = 1,155 min	3,837	1–7 d
8	84	MOF	MOFabric-19% (PVDF/UiO-66-NH_2)	GD	W0-B0	$t_{1/2}$ = 161 min	535	6–24 h
9	84	MOF	MOFabric-33% (PVDF/UiO-66-NH_2)	GD	W0-B0	$t_{1/2}$ = 131 min	435	6–24 h
10	81	MOF	MOF-808/BPEIH/fiber (dried 2 d under hood)	DMNP	W0-B0	$t_{1/2}$ = 20 min	66.4	1–6 h
11	110	Metal oxide	AP-Al_2O_3	GD	W1-B0	$t_{1/2}$ = 1.8 h	359	1–6 h
12	110	Metal oxide	AP-Al_2O_3	VX	W1-B0	$t_{1/2}$ > 6.4 d	> 30,615	> 7 d
13	106	Metal oxide	nMgO (AP-MgO)	GD	W1-B0	$t_{1/2}$ = 28 min	93	1–6 h
14	106	Metal oxide	nMgO (AP-MgO)	VX	W1-B0	$t_{1/2}$ = 68 h	13,553	> 7 d
15	108	Metal oxide	nCaO (AP-CaO)	GD	W1-B0	$t_{1/2}$ = 4.5 h	897	6–24 h
16	108	Metal oxide	nCaO (AP-CaO)	VX	W1-B0	$t_{1/2}$ = 93 h	18,536	> 7 d
17	109	Metal oxide	γ-Al_2O_3 (conventional alumina)	GD	W1-B0	$t_{1/2}$ = 1.8 h	359	1–6 h
18	109	Metal oxide	γ-Al_2O_3 (conventional alumina)	VX	W1-B0	$t_{1/2}$ = 9.2 d	44,009	> 7 d
19	109	Metal oxide	γ-Al_2O_3 (conventional alumina)	VX	W1-B0	$t_{1/2}$ = 6.8 d	32,528	> 7 d
20	111	Metal oxide	Nanotubular titania (NTT)	VX	W1-B0	$t_{1/2}$ = 24.7 min	82.1	1–6 h
21	97	Metal oxide	$nTiO_2$ (30 wt% H_2O)	VX	W1-B0	$t_{1/2}$ = 2 min	6.64	≤ 1 h
22	97	Metal oxide	$nTiO_2$ (no wax, 25 wt% H_2O)	GD	W1-B0	$t_{1/2}$ = 1.3 h	259	1–6 h

23	97	Metal oxide	$nTiO_2$ (no wax, 25 wt% H_2O)	VX	W1-B0	$t_{1/2}$ = 8 min	26.6	≤ 1 h
24	50	Metal hydroxide / oxide-like	$Zr(OH)_4$	GD	W1-B0	$t_{1/2}$ = 8.7 min	28.9	≤ 1 h
25	50	Metal hydroxide / oxide-like	$Zr(OH)_4$	VX	W1-B0	$t_{1/2}$ < 1 min	< 3.32	≤ 1 h
26	98	Metal hydroxide / oxide-like	$Zr(OH)_4$ (air, ~50% RH)	DMMP	W1-B0	k_{obs} = 0.00779 min^{-1}	296	1–6 h
27	98	Metal hydroxide / oxide-like	$Zr(OH)_4$ (humid N_2, 100% RH)	DMMP	W1-B0	k_{obs} = 0.0121 min^{-1}	190	1–6 h
28	61	Zeolites & porous silicates	AgY zeolite	VX	W1-B0	$t_{1/2}$ > 6.6 h	> 1,315	6–24 h
29	61	Zeolites & porous silicates	NaY zeolite	VX	W1-B0	$t_{1/2}$ > 5 d	> 23,918	> 7 d
30	76	Carbon-based materials & composites	Activated carbon (S-AC), humidified (RH 75%)	GB	W1-B0	$t_{1/2}$ = 4 d	19,134	> 7 d
31	76	Carbon-based materials & composites	Activated carbon (S-AC), humidified (RH 75%)	VX	W1-B0	$t_{1/2}$ = 14 d	66,970	> 7 d
32	77	MOF	NU-1000	GD	W1-B0	$t_{1/2}$ = 36 min	120	1–6 h
33	81	MOF	MOF-808/BPEIH powder	DMNP	W1-B0	$t_{1/2}$ = 1 min	3.32	≤ 1 h
34	81	MOF	MOF-808/BPEIH powder	DEMP (VX simulant)	W1-B0	$t_{1/2}$ = 1 min	3.32	≤ 1 h
35	81	MOF	MOF-808/BPEIH/fiber	DMNP	W1-B0	$t_{1/2}$ = 1 min	3.32	≤ 1 h
36	83	MOF	UiO-67-$N(Me)_2$	VX	W2-B0	$t_{1/2}$ = 7 min	23.3	≤ 1 h
37	92	POM	$\{Gd_3(HPO_3)Sb_3W_{28}\}$ in D_2O (pD 7, no buffer)	DMNP	W2-B0	k_{obs} = 0.000797 min^{-1}	2,889	1–7 d
38	92	POM	$\{Y_3(HPO_3)Sb_3W_{28}\}$ in D_2O (pD 7, no buffer)	DMNP	W2-B0	k_{obs} = 0.000923 min^{-1}	2,495	1–7 d
39	92	POM	$\{Yb_3(HPO_3)Sb_3W_{28}\}$ in D_2O (pD 7, no buffer)	DMNP	W2-B0	k_{obs} = 0.00128 min^{-1}	1,800	1–7 d
40	92	POM	$\{SbW_9\}$ lacunary precursor in D_2O (pD 7, no buffer)	DMNP	W2-B0	k_{obs} = 5.63e-05 min^{-1}	40,898	> 7 d
41	83	MOF	UiO-67-$N(Me)_2$	VX	W2-B2	$t_{1/2}$ = 1.8 min	5.98	≤ 1 h
42	83	MOF	UiO-67-NH_2	VX	W2-B2	$t_{1/2}$ = 6 min	19.9	≤ 1 h
43	83	MOF	UiO-67	VX	W2-B2	$t_{1/2}$ = 7.9 min	26.2	≤ 1 h
44	83	MOF	UiO-66	VX	W2-B2	$t_{1/2}$ = 90 min	299	1–6 h
45	77	MOF	NU-1000	DMNP	W2-B2	$t_{1/2}$ = 15 min	49.8	≤ 1 h

46	77	MOF	NU-1000-dehyd	DMNP	W2-B2	$t_{1/2}$ = 1.5 min	4.98	≤ 1 h
47	77	MOF	NU-1000	GD	W2-B2	$t_{1/2}$ = 3 min	9.97	≤ 1 h
48	86	MOF	MOF-808 6.5%/PET	DMNP	W2-B2	$t_{1/2}$ ≤ 0.5 min	≤ 1.66	≤ 1 h
49	86	MOF	MOF-808 12%/PET	DMNP	W2-B2	$t_{1/2}$ ≤ 1 min	≤ 3.32	≤ 1 h
50	86	MOF	MOF-808 22%/PET	DMNP	W2-B2	$t_{1/2}$ = 3 min	9.97	≤ 1 h
51	86	MOF	UiO-66-NH_2/PET (8% loading)	DMNP	W2-B2	$t_{1/2}$ = 5 min	16.6	≤ 1 h
52	86	MOF	MOF-808/PET	GD	W2-B2	$t_{1/2}$ = 2 min	6.64	≤ 1 h
53	86	MOF	UiO-66-NH_2/PET	GD	W2-B2	$t_{1/2}$ = 8 min	26.6	≤ 1 h
54	92	POM	{$Gd_3(HPO_3)Sb_3W_{28}$} in 125 mM Tris-HCl (pD 7)	DMNP	W2-B1	k_{obs} = 0.000304 min^{-1}	7,574	1–7 d
55	92	POM	{$Y_3(HPO_3)Sb_3W_{28}$} in 125 mM Tris-HCl (pD 7)	DMNP	W2-B1	k_{obs} = 0.000446 min^{-1}	5,163	1–7 d
56	92	POM	{$Yb_3(HPO_3)Sb_3W_{28}$} in 125 mM Tris-HCl (pD 7)	DMNP	W2-B1	k_{obs} = 0.000779 min^{-1}	2,956	1–7 d
57	92	POM	{SbW_9} lacunary precursor in 125 mM Tris-HCl (pD 7)	DMNP	W2-B1	k_{obs} = 0.00051 min^{-1}	4,515	1–7 d
58	104	Zeolites & porous silicates	P-SBA-15	DMNP	W2-B2	$t_{1/2}$ > 220 min	> 731	6–24 h
59	104	Zeolites & porous silicates	Zr_{12}-acetic acid-P-SBA-15	DMNP	W2-B2	$t_{1/2}$ = 69 min	229	1–6 h
60	104	Zeolites & porous silicates	H-SO_3H-SBA-15	DMNP	W2-B2	$t_{1/2}$ > 1,080 min	> 3,588	1–7 d
61	104	Zeolites & porous silicates	Zr-SBA-15	DMNP	W2-B2	$t_{1/2}$ > 550 min	> 1,827	1–7 d
62	104	Zeolites & porous silicates	SBA-15 + Zr_{12}-acetic acid	DMNP	W2-B2	$t_{1/2}$ = 69 min	229	1–6 h
63	104	Zeolites & porous silicates	Zr_6-acetic acid-L-SO_3H-SBA-15	DMNP	W2-B2	$t_{1/2}$ = 89 min	296	1–6 h
64	104	Zeolites & porous silicates	Zr_6-propanoic acid-L-SO_3H-SBA-15	DMNP	W2-B2	$t_{1/2}$ = 197 min	654	6–24 h
65	104	Zeolites & porous silicates	Zr_6-benzoic acid-L-SO_3H-SBA-15	DMNP	W2-B2	$t_{1/2}$ = 407 min	1,352	6–24 h
66	104	Zeolites & porous silicates	Zr_6-formic acid-L-SO_3H-SBA-15	DMNP	W2-B2	$t_{1/2}$ = 717 min	2,382	1–7 d
67	104	Zeolites & porous silicates	Zr_{12}-formic acid-L-SO_3H-SBA-15	DMNP	W2-B2	$t_{1/2}$ = 85 min	282	1–6 h
68	104	Zeolites & porous silicates	Zr_{12}-acetic acid-L-SO_3H-SBA-15	DMNP	W2-B2	$t_{1/2}$ = 221 min	734	6–24 h

69	104	Zeolites & porous silicates	Zr_{12}-propanoic acid-L-SO_3H-SBA-15	DMNP	W2-B2	$t_{1/2}$ = 283 min	940	6–24 h
70	44	MOF	MOF-808 (6-connected)	DMNP	W2-B2	$t_{1/2}$ ≤ 0.5 min	≤ 1.66	≤ 1 h
71	84	MOF	UiO-66-NH_2 powder	DMNP	W2-B2	$t_{1/2}$ = 4.4 min	14.6	≤ 1 h
72	84	MOF	MOFabric-7% (PVDF/UiO-66-NH_2)	DMNP	W2-B2	$t_{1/2}$ = 210 min	698	6–24 h
73	84	MOF	MOFabric-13% (PVDF/UiO-66-NH_2)	DMNP	W2-B2	$t_{1/2}$ = 75 min	249	1–6 h
74	84	MOF	MOFabric-19% (PVDF/UiO-66-NH_2)	DMNP	W2-B2	$t_{1/2}$ = 36 min	120	1–6 h
75	84	MOF	MOFabric-33% (PVDF/UiO-66-NH_2)	DMNP	W2-B2	$t_{1/2}$ = 12 min	39.9	≤ 1 h
76	85	MOF	UiO-66-NH_2 powder (1.5 mg; 0.9 µmol catalyst)	DMNP	W2-B2	$t_{1/2}$ = 36.5 min	121	1–6 h
77	85	MOF	UiO-66-NH_2 powder (2.6 mg; 1.5 µmol catalyst)	DMNP	W2-B2	$t_{1/2}$ = 6.5 min	21.6	≤ 1 h
78	85	MOF	UiO-66-NH_2 powder (5.6 mg; 3.3 µmol catalyst)	DMNP	W2-B2	$t_{1/2}$ = 2.5 min	8.3	≤ 1 h
79	85	MOF	PP/ZnO + UiO-66-NH_2 assembly (10.0 mg total; 1.8 µmol catalyst; 30.7 wt% MOF)	DMNP	W2-B2	$t_{1/2}$ = 6.5 min	21.6	≤ 1 h
80	85	MOF	PP/ZnO + UiO-66-NH_2 assembly (17.6 mg total; 3.2 µmol catalyst; 30.7 wt% MOF)	DMNP	W2-B2	$t_{1/2}$ = 2.8 min	9.3	≤ 1 h

Table S2. Experimental conditions and loading-adjusted apparent kinetic descriptors for the entries in Table S1, including EA-2192 outcomes for VX-containing entries. NC, not calculated; NR, not reported in the original source; N/A, non-VX entry. For EA-2192 outcomes, “Not detected” means that EA-2192 was not detected under the reported analytical conditions, whereas “Not assessed” means that EA-2192 formation was not evaluated or not reported.

No.	Ref.	Specific Decontaminant	W/B-class	Experimental condition	Agent loading, n_{agent}/m_{decon} (mmol/g)	k_{app} (min^{-1})	Loading-adjusted apparent descriptor	EA-2192 outcome	NC basis
1	98	$Zr(OH)_4$ (dry CO_2 pre-dose)	W0-B0	$Zr(OH)_4$ ≈ 200 mg; DMMP = 100 µL ≈ 923 µmol; no bulk V	4.61 mmol g^{-1}	$1.37{\times}10^{-3}$ min^{-1}	$r_{app,m}$ = 6.32 µmol g^{-1} min^{-1}	N/A	
2	98	$Zr(OH)_4$ (dry N_2-purged)	W0-B0	$Zr(OH)_4$ ≈ 200 mg; DMMP = 100 µL ≈ 923 µmol; no bulk V	4.61 mmol g^{-1}	$7.08{\times}10^{-3}$ min^{-1}	$r_{app,m}$ = 32.7 µmol g^{-1} min^{-1}	N/A	
3	70	TiO_2/MCM reactive adsorbent	W0-B0	TiO_2/MCM = 50 mg; V = 4 mL; GB = 4 mg ≈ 28.6 µmol; hexane suspension	0.571 mmol g^{-1}	$2.17{\times}10^{-1}$ min^{-1}	$k_{app}V_{rxn}/m_{decon}$ = $1.73{\times}10^{-2}$ L g^{-1} min^{-1}	N/A	
4	76	Activated carbon (S-AC), dry	W0-B0	S-AC ≈ 40 mg; VX = 2 µL ≈ 7.56 µmol; no bulk V	0.189	$3.44{\times}10^{-5}$	$r_{app,m}$ = $6.49{\times}10^{-3}$ µmol g^{-1} min^{-1}	Not assessed	
5	84	UiO-66-NH_2 powder	W0-B0	GD = 2.6 µL ≈ 14.6 µmol; m_{cat} NR	NC	$2.20{\times}10^{-3}$ min^{-1}	NC	N/A	m_{decon} NR
6	84	MOFabric-7% (PVDF/UiO-66-NH_2)	W0-B0	GD = 2.6 µL ≈ 14.6 µmol; 1 cm^2 swatch; m_{MOF} NR	NC	$4.29{\times}10^{-4}$ min^{-1}	NC	N/A	active MOF mass NR
7	84	MOFabric-13% (PVDF/UiO-66-NH_2)	W0-B0	GD = 2.6 µL ≈ 14.6 µmol; 1 cm^2 swatch; m_{MOF} NR	NC	$6.00{\times}10^{-4}$ min^{-1}	NC	N/A	active MOF mass NR
8	84	MOFabric-19% (PVDF/UiO-66-NH_2)	W0-B0	GD = 2.6 µL ≈ 14.6 µmol; 1 cm^2 swatch; m_{MOF} NR	NC	$4.31{\times}10^{-3}$ min^{-1}	NC	N/A	active MOF mass NR
9	84	MOFabric-33% (PVDF/UiO-66-NH_2)	W0-B0	GD = 2.6 µL ≈ 14.6 µmol; 1 cm^2 swatch; m_{MOF} NR	NC	$5.29{\times}10^{-3}$ min^{-1}	NC	N/A	active MOF mass NR
10	81	MOF-808/BPEIH/fiber (dried 2 d under hood)	W0-B0	m_MOF ≈ 2.2 mg; DMNP = 25 µmol; dried 2 d under hood	11.4 mmol g_{MOF}^{-1}	$3.47{\times}10^{-2}$ min^{-1}	$r_{app,m}$ = 394 µmol g_{MOF}^{-1} min^{-1}	N/A	
11	110	AP-Al_2O_3	W1-B0	5 wt% GD; exact m_{cat} NR; S_{BET} = 273.6 m^2 g^{-1}	0.274	$6.42{\times}10^{-3}$	$r_{app,m}$ = 1.76 µmol g^{-1} min^{-1}	N/A	
12	110	AP-Al_2O_3	W1-B0	5 wt% VX; exact m_{cat} NR; S_{BET} = 273.6 m^2 g^{-1}	0.187	$<7.52{\times}10^{-5}$	$r_{app,m}$ < $1.41{\times}10^{-2}$ µmol g^{-1} min^{-1}	Not detected	
13	106	nMgO (AP-MgO)	W1-B0	AP-MgO = 106 mg; GD = 6 µL ≈ 33.6 µmol; S_{BET} = 344 m^2 g^{-1}	0.317	$2.48{\times}10^{-2}$	$r_{app,m}$ = 7.85 µmol g^{-1} min^{-1}	N/A	

14	106	nMgO (AP-MgO)	W1-B0	AP-MgO = 104 mg; VX = 6 µL ≈ 22.7 µmol; S_{BET} = 344 m^2 g^{-1}	0.218	1.70×10^{-4}	$r_{app,m}$ = 3.70×10^{-2} µmol g^{-1} min^{-1}	Not detected	
15	108	nCaO (AP-CaO)	W1-B0	AP-CaO = 115.7 mg; GD = 6 µL ≈ 33.6 µmol	0.29	2.57×10^{-3}	$r_{app,m}$ = 0.745 µmol g^{-1} min^{-1}	N/A	
16	108	nCaO (AP-CaO)	W1-B0	AP-CaO = 110.6 mg; VX = 6 µL ≈ 22.7 µmol	0.205	1.24×10^{-4}	$r_{app,m}$ = 2.55×10^{-2} µmol g^{-1} min^{-1}	Not detected	
17	109	γ-Al_2O_3 (conventional alumina)	W1-B0	GD = 3–16 µL; 1.5–5 wt%; S_{BET} = 400 m^2 g^{-1}	0.082–0.274	6.42×10^{-3}	$r_{app,m}$ = 0.528–1.76 µmol g^{-1} min^{-1}	N/A	
18	109	γ-Al_2O_3 (conventional alumina)	W1-B0	VX = 15 µL; ca. 5 wt%; m_{cat} back-calculated ≈ 303 mg	0.187	5.23×10^{-5}	$r_{app,m}$ = 9.78×10^{-3} µmol g^{-1} min^{-1}	Not assessed	
19	109	γ-Al_2O_3 (conventional alumina)	W1-B0	VX = 3.5 µL; ca. 1.5 wt%; m_{cat} back-calculated ≈ 236 mg	0.0561	7.08×10^{-5}	$r_{app,m}$ = 3.97×10^{-3} µmol g^{-1} min^{-1}	Not assessed	
20	111	Nanotubular titania (NTT)	W1-B0	NTT ≈ 0.15 g; VX = 8 µL ≈ 30.2 µmol; H_2O = 40 µL (20 wt%); no bulk V	0.201	2.81×10^{-2}	$r_{app,m}$ =5.65 µmol g^{-1} min^{-1}	Not assessed	
21	97	$nTiO_2$ (30 wt% H_2O)	W1-B0	$nTiO_2$ m_{cat} NR; VX = 5 µL ≈ 18.9 µmol; S_{BET} = 489 m^2 g^{-1}	NC	3.47×10^{-1}	NC	Not assessed	m_{decon} NR
22	97	$nTiO_2$ (no wax, 25 wt% H_2O)	W1-B0	$nTiO_2$ m_{cat} NR; GD = 5 µL ≈ 28.0 µmol; S_{BET} = 489 m^2 g^{-1}	NC	8.89×10^{-3}	NC	N/A	m_{decon} NR
23	97	$nTiO_2$ (no wax, 25 wt% H_2O)	W1-B0	$nTiO_2$ m_{cat} NR; VX = 5 µL ≈ 18.9 µmol; S_{BET} = 489 m^2 g^{-1}	NC	8.66×10^{-2}	NC	Not assessed	m_{decon} NR
24	50	$Zr(OH)_4$	W1-B0	$Zr(OH)_4$ = 0.265 g; GD = 5 µL ≈ 28.0 µmol	0.106	7.97×10^{-2}	$r_{app,m}$ = 8.42 µmol g^{-1} min^{-1}	N/A	
25	50	$Zr(OH)_4$	W1-B0	$Zr(OH)_4$ = 0.234 g; VX = 20 µL ≈ 75.6 µmol	0.323	> 6.93×10^{-1}	$r_{app,m}$ > 224 µmol g^{-1} min^{-1}	Not detected	
26	98	$Zr(OH)_4$ (air, ~50% RH)	W1-B0	$Zr(OH)_4$ ≈ 200 mg; DMMP = 100 µL ≈ 923 µmol; no bulk V	4.61 mmol g^{-1}	7.79×10^{-3} min^{-1}	$r_{app,m}$ = 35.9 µmol g^{-1} min^{-1}	N/A	
27	98	$Zr(OH)_4$ (humid N_2, 100% RH)	W1-B0	$Zr(OH)_4$ ≈ 200 mg; DMMP = 100 µL ≈ 923 µmol; no bulk V	4.61 mmol g^{-1}	1.21×10^{-2} min^{-1}	$r_{app,m}$ = 55.8 µmol g^{-1} min^{-1}	N/A	
28	61	AgY zeolite	W1-B0	AgY = 0.2761 g; VX = 15 µL ≈ 56.7 µmol; 13 wt% water	0.205 mmol g^{-1}	<1.75×10^{-3} min^{-1}	$r_{app,m}$ < 0.359 µmol g^{-1} min^{-1}	Not detected	

29	61	NaY zeolite	W1-B0	NaY = 0.1987 g; VX = 11 μL ≈ 41.6 μmol; 20 wt% water	0.209 mmol g^{-1}	<9.63×10^{-5} min^{-1}	$r_{app,m} < 2.01\times10^{-2}$ μmol g^{-1} min^{-1}	Not detected	
30	76	Activated carbon (S-AC), humidified (RH 75%)	W1-B0	S-AC ≈ 40 mg; GB = 2 μL ≈ 15.6 μmol; no bulk V	0.389	1.20×10^{-4}	$r_{app,m} = 4.68\times10^{-2}$ μmol g^{-1} min^{-1}	N/A	
31	76	Activated carbon (S-AC), humidified (RH 75%)	W1-B0	S-AC ≈ 40 mg; VX = 2 μL ≈ 7.56 μmol; no bulk V	0.189	3.44×10^{-5}	$r_{app,m} = 6.49\times10^{-3}$ μmol g^{-1} min^{-1}	Not detected	
32	77	NU-1000	W1-B0	NU-1000 = 30.9 mg; GD = 5 μL ≈ 28.0 μmol; solid-state test at 50% RH	0.906 mmol g^{-1}	1.93×10^{-2} min^{-1}	$r_{app,m}$ = 17.4 μmol g^{-1} min^{-1}	N/A	
33	81	MOF-808/BPEIH powder	W1-B0	m_{MOF} = 2.2 mg; composite contains BPEIH; DMNP = 25 μmol	11.4 mmol g_{MOF}^{-1}	6.93×10^{-1} min^{-1}	$r_{app,m} = 7.88\times10^{3}$ μmol g_{MOF}^{-1} min^{-1}	N/A	
34	81	MOF-808/BPEIH powder	W1-B0	m_{MOF} = 2.2 mg; composite contains BPEIH; DEMP = 25 μmol	11.4 mmol g_{MOF}^{-1}	6.93×10^{-1} min^{-1}	$r_{app,m} = 7.88\times10^{3}$ μmol g_{MOF}^{-1} min^{-1}	N/A	
35	81	MOF-808/BPEIH/fiber	W1-B0	1×1 cm fiber; contains 1.5 μmol MOF-808 ≈ 2.2 mg; DMNP = 25 μmol	11.4 mmol g_{MOF}^{-1}	6.93×10^{-1} min^{-1}	$r_{app,m} = 7.88\times10^{3}$ μmol g_{MOF}^{-1} min^{-1}	N/A	
36	83	UiO-67-N(Me)$_2$	W2-B0	m_cat = 2.5 mg; V = 0.75 mL; VX = 14.7 μmol; pure water, no external base/buffer	5.88	9.90×10^{-2}	$k_{app}V_{rxn}/m_{decon} = 2.97\times10^{-2}$ L g^{-1} min^{-1}	Not detected	
37	92	{$Gd_3(HPO_3)Sb_3W_{28}$} in D_2O (pD 7, no buffer)	W2-B0	DMNP concentration reported; POM concentration likely comparable; mass/total V NR	NC	7.97×10^{-4} min^{-1}	NC	N/A	concentration basis only; mass/volume conditions NR
38	92	{$Y_3(HPO_3)Sb_3W_{28}$} in D_2O (pD 7, no buffer)	W2-B0	DMNP concentration reported; POM concentration likely comparable; mass/total V NR	NC	9.23×10^{-4} min^{-1}	NC	N/A	concentration basis only; mass/volume conditions NR
39	92	{$Yb_3(HPO_3)Sb_3W_{28}$} in D_2O (pD 7, no buffer)	W2-B0	DMNP concentration reported; POM concentration likely comparable; mass/total V NR	NC	1.28×10^{-3} min^{-1}	NC	N/A	concentration basis only; mass/volume conditions NR
40	92	{SbW_9} lacunary precursor in D_2O (pD 7, no buffer)	W2-B0	DMNP concentration reported; POM concentration likely comparable; mass/total V NR	NC	5.63×10^{-5} min^{-1}	NC	N/A	concentration basis only; mass/volume conditions NR
41	83	UiO-67-N(Me)$_2$	W2-B2	m_{cat} = 2.5 mg; V ≈ 0.75 mL; VX = 14.7 μmol; 0.5 M NEM, pH 10	5.88	3.85×10^{-1}	$k_{app}V_{rxn}/m_{decon} = 1.16\times10^{-1}$ L g^{-1} min^{-1}	Not detected	
42	83	UiO-67-NH$_2$	W2-B2	m_{cat} = 2.5 mg; V ≈ 0.75 mL; VX = 14.7 μmol	5.88	1.16×10^{-1}	$k_{app}V_{rxn}/m_{decon} = 3.47\times10^{-2}$ L g^{-1} min^{-1}	Not detected	
43	83	UiO-67	W2-B2	m_{cat} = 2.5 mg; V ≈ 0.75 mL; VX = 14.7 μmol	5.88	8.77×10^{-2}	$k_{app}V_{rxn}/m_{decon} = 2.63\times10^{-2}$ L g^{-1} min^{-1}	Not detected	

44	83	UiO-66	W2-B2	m_{cat} = 2.5 mg; V ≈ 0.75 mL; VX = 14.7 μmol	5.88	7.70×10^{-3}	$k_{app}V_{rxn}/m_{decon}$ = 2.31×10^{-3} L g^{-1} min^{-1}	Not detected	
45	77	NU-1000	W2-B2	NU-1000 = 3.0 mg; V ≈ 1.0 mL; DMNP = 25 μmol	8.33 mmol g^{-1}	4.62×10^{-2} min^{-1}	$k_{app}V_{rxn}/m_{decon}$ = 1.54×10^{-2} L g^{-1} min^{-1}	N/A	
46	77	NU-1000-dehyd	W2-B2	NU-1000-dehyd = 3.0 mg; V ≈ 1.0 mL; DMNP = 25 μmol	8.33 mmol g^{-1}	4.62×10^{-1} min^{-1}	$k_{app}V_{rxn}/m_{decon}$ = 1.54×10^{-1} L g^{-1} min^{-1}	N/A	
47	77	NU-1000	W2-B2	NU-1000 = 3.3 mg; V ≈ 0.747 mL; GD = 2.6 μL ≈ 14.6 μmol	4.41 mmol g^{-1}	2.31×10^{-1} min^{-1}	$k_{app}V_{rxn}/m_{decon}$ = 5.23×10^{-2} L g^{-1} min^{-1}	N/A	
48	86	MOF-808 6.5%/PET	W2-B2	Catalyst = 1.5 μmol; DMNP = 25 μmol; MOF-808 6.5%/PET	NC	≥ 1.39 min^{-1}	NC	N/A	active MOF mass NR
49	86	MOF-808 12%/PET	W2-B2	Catalyst = 1.5 μmol; DMNP = 25 μmol; MOF-808 12%/PET	NC	≥ 6.93×10^{-1} min^{-1}	NC	N/A	active MOF mass NR
50	86	MOF-808 22%/PET	W2-B2	Catalyst = 1.5 μmol; DMNP = 25 μmol; MOF-808 22%/PET	NC	2.31×10^{-1} min^{-1}	NC	N/A	active MOF mass NR
51	86	UiO-66-NH_2/PET (8% loading)	W2-B2	Catalyst = 1.5 μmol; DMNP = 25 μmol; UiO-66-NH_2/PET, 8% loading	NC	1.39×10^{-1} min^{-1}	NC	N/A	active MOF mass NR
52	86	MOF-808/PET	W2-B2	Catalyst = 1.5 μmol; GD likely 25 μmol basis; MOF-808/PET	NC	3.47×10^{-1} min^{-1}	NC	N/A	active MOF mass NR; n_{agent} inferred
53	86	UiO-66-NH_2/PET	W2-B2	Catalyst = 1.5 μmol; GD likely 25 μmol basis; UiO-66-NH_2/PET	NC	8.66×10^{-2} min^{-1}	NC	N/A	active MOF mass NR; n_{agent} inferred
54	92	{$Gd_3(HPO_3)Sb_3W_{28}$} in 125 mM Tris-HCl (pD 7)	W2-B1	DMNP = 4.2 mM; POM = 2.5 mM reported for representative test	NC	3.04×10^{-4} min^{-1}	NC	N/A	concentration basis only; mass/volume conditions NR
55	92	{$Y_3(HPO_3)Sb_3W_{28}$} in 125 mM Tris-HCl (pD 7)	W2-B1	DMNP = 4.2 mM; POM = 2.5 mM reported for representative test	NC	4.46×10^{-4} min^{-1}	NC	N/A	concentration basis only; mass/volume conditions NR
56	92	{$Yb_3(HPO_3)Sb_3W_{28}$} in 125 mM Tris-HCl (pD 7)	W2-B1	DMNP = 4.2 mM; POM = 2.5 mM reported for representative test	NC	7.79×10^{-4} min^{-1}	NC	N/A	concentration basis only; mass/volume conditions NR
57	92	{SbW_9} lacunary precursor in 125 mM Tris-HCl (pD 7)	W2-B1	DMNP = 4.2 mM; POM = 2.5 mM reported for representative test	NC	5.10×10^{-4} min^{-1}	NC	N/A	concentration basis only; mass/volume conditions NR
58	104	P-SBA-15	W2-B2	Catalyst ≈ 8.65 mg; V ≈ 1.054 mL; DMNP = 25 μmol	2.89	<3.15×10^{-3} min^{-1}	$k_{app}V_{rxn}/m_{decon}$ < 3.84×10^{-4} L g^{-1} min^{-1}	N/A	

59	104	Zr_{12}-acetic acid-P-SBA-15	W2-B2	Catalyst ≈ 8.65 mg; V ≈ 1.054 mL; DMNP = 25 µmol	2.89	1.00×10^{-2} min^{-1}	$k_{app}V_{rxn}/m_{decon}$ $= 1.22\times10^{-3}$ L g^{-1} min^{-1}	N/A	
60	104	H-SO_3H-SBA-15	W2-B2	Catalyst ≈ 8.65 mg; V ≈ 1.054 mL; DMNP = 25 µmol	2.89	$<6.42\times10^{-4}$ min^{-1}	$k_{app}V_{rxn}/m_{decon}$ $< 7.82\times10^{-5}$ L g^{-1} min^{-1}	N/A	
61	104	Zr-SBA-15	W2-B2	Catalyst ≈ 8.65 mg; V ≈ 1.054 mL; DMNP = 25 µmol	2.89	$<1.26\times10^{-3}$ min^{-1}	$k_{app}V_{rxn}/m_{decon}$ $< 1.54\times10^{-4}$ L g^{-1} min^{-1}	N/A	
62	104	SBA-15 + Zr_{12}-acetic acid	W2-B2	Catalyst ≈ 8.65 mg; V ≈ 1.054 mL; DMNP = 25 µmol	2.89	1.00×10^{-2} min^{-1}	$k_{app}V_{rxn}/m_{decon}$ $= 1.22\times10^{-3}$ L g^{-1} min^{-1}	N/A	
63	104	Zr_6-acetic acid-L-SO_3H-SBA-15	W2-B2	Catalyst ≈ 8.65 mg; V ≈ 1.054 mL; DMNP = 25 µmol	2.89	7.79×10^{-3} min^{-1}	$k_{app}V_{rxn}/m_{decon}$ $= 9.49\times10^{-4}$ L g^{-1} min^{-1}	N/A	
64	104	Zr_6-propanoic acid-L-SO_3H-SBA-15	W2-B2	Catalyst ≈ 8.65 mg; V ≈ 1.054 mL; DMNP = 25 µmol	2.89	3.52×10^{-3} min^{-1}	$k_{app}V_{rxn}/m_{decon}$ $= 4.29\times10^{-4}$ L g^{-1} min^{-1}	N/A	
65	104	Zr_6-benzoic acid-L-SO_3H-SBA-15	W2-B2	Catalyst ≈ 8.65 mg; V ≈ 1.054 mL; DMNP = 25 µmol	2.89	1.70×10^{-3} min^{-1}	$k_{app}V_{rxn}/m_{decon}$ $= 2.08\times10^{-4}$ L g^{-1} min^{-1}	N/A	
66	104	Zr_6-formic acid-L-SO_3H-SBA-15	W2-B2	Catalyst ≈ 8.65 mg; V ≈ 1.054 mL; DMNP = 25 µmol	2.89	9.67×10^{-4} min^{-1}	$k_{app}V_{rxn}/m_{decon}$ $= 1.18\times10^{-4}$ L g^{-1} min^{-1}	N/A	
67	104	Zr_{12}-formic acid-L-SO_3H-SBA-15	W2-B2	Catalyst ≈ 8.65 mg; V ≈ 1.054 mL; DMNP = 25 µmol	2.89	8.15×10^{-3} min^{-1}	$k_{app}V_{rxn}/m_{decon}$ $= 9.94\times10^{-4}$ L g^{-1} min^{-1}	N/A	
68	104	Zr_{12}-acetic acid-L-SO_3H-SBA-15	W2-B2	Catalyst ≈ 8.65 mg; V ≈ 1.054 mL; DMNP = 25 µmol	2.89	3.14×10^{-3} min^{-1}	$k_{app}V_{rxn}/m_{decon}$ $= 3.82\times10^{-4}$ L g^{-1} min^{-1}	N/A	
69	104	Zr_{12}-propanoic acid-L-SO_3H-SBA-15	W2-B2	Catalyst ≈ 8.65 mg; V ≈ 1.054 mL; DMNP = 25 µmol	2.89	2.45×10^{-3} min^{-1}	$k_{app}V_{rxn}/m_{decon}$ $= 2.98\times10^{-4}$ L g^{-1} min^{-1}	N/A	
70	44	MOF-808 (6-connected)	W2-B2	MOF-808 ≈ 0.5 mg; V ≈ 1.0 mL; DMNP = 25 µmol	50.0 mmol g^{-1}	≥ 1.39 min^{-1}	$k_{app}V_{rxn}/m_{decon}$ ≥ 2.77 L g^{-1} min^{-1}	N/A	
71	84	UiO-66-NH_2 powder	W2-B2	UiO-66-NH_2 powder; DMNP; m_{cat} and n_{DMNP} NR in main text	NC	1.58×10^{-1} min^{-1}	NC	N/A	m_{decon} and n_{agent} NR
72	84	MOFabric-7% (PVDF/UiO-66-NH_2)	W2-B2	MOFabric-7%; DMNP; m_{cat} and n_{DMNP} NR in main text	NC	3.30×10^{-3} min^{-1}	NC	N/A	m_{decon} and n_{agent} NR
73	84	MOFabric-13% (PVDF/UiO-66-NH_2)	W2-B2	MOFabric-13%; DMNP; m_{cat} and n_{DMNP} NR in main text	NC	9.24×10^{-3} min^{-1}	NC	N/A	m_{decon} and n_{agent} NR

74	84	MOFabric-19% (PVDF/UiO-66-NH_2)	W2-B2	MOFabric-19%; DMNP; m_{cat} and n_{DMNP} NR in main text	NC	1.93×10^{-2} min^{-1}	NC	N/A	m_{decon} and n_{agent} NR
75	84	MOFabric-33% (PVDF/UiO-66-NH_2)	W2-B2	MOFabric-33%; DMNP; m_{cat} and n_{DMNP} NR in main text	NC	5.78×10^{-2} min^{-1}	NC	N/A	m_{decon} and n_{agent} NR
76	85	UiO-66-NH_2 powder (1.5 mg; 0.9 µmol catalyst)	W2-B2	UiO-66-NH_2 = 1.5 mg; V = 1 mL; DMNP = 25 µmol	16.7 mmol g^{-1}	1.90×10^{-2} min^{-1}	$k_{app}V_{rxn}/m_{decon}$ = 1.27×10^{-2} L g^{-1} min^{-1}	N/A	
77	85	UiO-66-NH_2 powder (2.6 mg; 1.5 µmol catalyst)	W2-B2	UiO-66-NH_2 = 2.6 mg; V = 1 mL; DMNP = 25 µmol	9.62 mmol g^{-1}	1.07×10^{-1} min^{-1}	$k_{app}V_{rxn}/m_{decon}$ = 4.10×10^{-2} L g^{-1} min^{-1}	N/A	
78	85	UiO-66-NH_2 powder (5.6 mg; 3.3 µmol catalyst)	W2-B2	UiO-66-NH_2 = 5.6 mg; V = 1 mL; DMNP = 25 µmol	4.46 mmol g^{-1}	2.77×10^{-1} min^{-1}	$k_{app}V_{rxn}/m_{decon}$ = 4.95×10^{-2} L g^{-1} min^{-1}	N/A	
79	85	PP/ZnO + UiO-66-NH_2 assembly (10.0 mg total; 1.8 µmol catalyst; 30.7 wt% MOF)	W2-B2	Total fabric = 10.0 mg; active MOF ≈ 3.07 mg; V = 1 mL; DMNP = 25 µmol	8.14 mmol g_{MOF}^{-1}	1.07×10^{-1} min^{-1}	$k_{app}V_{rxn}/m_{decon}$ = 3.47×10^{-2} L g_{MOF}^{-1} min^{-1}	N/A	
80	85	PP/ZnO + UiO-66-NH_2 assembly (17.6 mg total; 3.2 µmol catalyst; 30.7 wt% MOF)	W2-B2	Total fabric = 17.6 mg; active MOF ≈ 5.40 mg; V = 1 mL; DMNP = 25 µmol	4.63 mmol g_{MOF}^{-1}	2.48×10^{-1} min^{-1}	$k_{app}V_{rxn}/m_{decon}$ = 4.58×10^{-2} L g_{MOF}^{-1} min^{-1}	N/A	
